# Ketazine‐Linked Covalent Organic Framework for Metal‐Free Electrocatalytic Nitrate‐to‐Ammonia Conversion

**DOI:** 10.1002/anie.6570629

**Published:** 2026-06-03

**Authors:** Islam E. Khalil, Ashadul Adalder, Badr Elkamash, Narad Barman, Darosch Asgari, Luoxing Xiang, Warisha Tahir, Franziska Hess, Ranjit Thapa, Adisak Boonchun, Uttam Kumar Ghorai, Prasenjit Das, Arne Thomas

**Affiliations:** ^1^ Department of Chemistry Functional Materials Technische Universität Berlin Berlin Germany; ^2^ Department of Industrial Chemistry & Applied Chemistry Swami Vivekananda Research Centre, Ramakrishna Mission Vidyamandira, Belur Math Howrah India; ^3^ Institute For Chemistry Technical University Berlin Berlin Germany; ^4^ Faculty of Science Department of Mathematics and Computer Science Alexandria University Alexandria Egypt; ^5^ Department of Physics SRM University – AP Amaravati Andhra Pradesh India; ^6^ Thomas Lord Department of Mechanical Engineering and Materials Science Duke University Durham North Carolina USA; ^7^ Centre for Computational and Integrative Sciences SRM University – AP Amaravati Andhra Pradesh India; ^8^ Faculty of Science Department of Physics Kasetsart University Bangkok Thailand; ^9^ Department of Chemistry Indian Institute of Technology Ropar SSB Block Rupnagar Punjab India; ^10^ Department of Chemistry, Chair of Macromolecular Chemistry Technische Universität München Garching Germany

**Keywords:** ammonia, covalent organic frameworks, electrocatalysis, ketazine linkage, metal‐free catalysis, nitrate reduction reaction (NO_3_RR)

## Abstract

The electrocatalytic nitrate reduction reaction (NO_3_RR) enables sustainable nitrate remediation and simultaneous ammonia synthesis as an alternative to the energy‐intensive Haber‐Bosch process. Recently, covalent organic frameworks (COFs) have garnered attention as NO_3_RR platform due to their intrinsic porosity and structural tunability, which enable active‐site engineering. However, current COF‐based systems largely rely on metal active sites, undermining their sustainability and obscuring the catalytic activity of the framework. Here, we introduce a metal‐free COF, that is highly active for NO_3_RR. The electrocatalyst is a fluorinated ketazine‐linked COF (F‐Ketazine COF), which incorporates electron‐withdrawing fluorine atoms that enhance π–π stacking interactions and promote efficient linkage formation. Synthesized via a rapid solvothermal reaction, the F‐Ketazine COF exhibits superior crystallinity and enhanced electrocatalytic activity compared to its non‐fluorinated analog (n‐Ketazine COF). Under neutral pH conditions, the F‐Ketazine COF achieved a Faradaic efficiency of 59.9% for NH_3_ and an NH_3_ yield rate of 1639.9 µmol h^−1^ mg_COF_^−1^ at −0.9 V versus RHE. To the best of our knowledge, this is the first example of a ketazine‐linked COF applied in electrocatalysis and the first a metal‐free COF for NO_3_RR. This work demonstrates the potential of fluorinated ketazine linkages in developing sustainable and efficient COF‐based electrocatalysts.

## Graphical abstract

A metal‐free fluorinated ketazine‐linked COF is introduced as an efficient electrocatalyst for nitrate reduction to ammonia under neutral conditions. Fluorine substitution enhances π–π stacking and crystallinity, enabling a Faradaic efficiency of 59.9% and an NH_3_ yield rate of 1639.9 µmol h^−1^ mg_COF_^−1^ at −0.9 V versus RHE. This establishes an unexplored metal‐free ketazine COF platform for NO_3_RR.

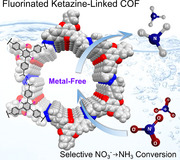

## 1 Introduction

Ammonia (NH_3_) plays a vital role in modern society, not only as a key industrial chemical but also as a potential hydrogen carrier and high‐energy‐density fuel in future energy systems [1]. Conventionally, NH_3_ is synthesized via the Haber‐Bosch process, which converts nitrogen (N_2_) and hydrogen (H_2_) into ammonia under harsh conditions of 400–500°C and 150–300 atm. Despite its effectiveness, this process is highly energy‐intensive and contributes significantly to global carbon emissions [2, 3, 4]. As a more sustainable alternative, electrocatalytic ammonia synthesis powered by renewable electricity has garnered increasing attention due to its environmentally friendly nature. Among possible nitrogen sources, nitrate (NO_3_^−^) offers practical advantages over molecular nitrogen: it is highly soluble in aqueous media and has a lower bond dissociation energy for the N═O bond (204 kJ mol^−1^) compared to the triple bond in N_2_ (945 kJ mol^−1^), making it more amenable to activation [5, 6, 7]. Furthermore, nitrate is a common contaminant in industrial and municipal wastewater, posing environmental risks. Consequently, the electrochemical nitrate reduction reaction (NO_3_RR) has emerged as a promising strategy for converting pollutants into valuable products like NH_3_. This reaction, however, involves a complex 8‐electron and 9‐proton transfer process (NO_3_^−^ + 8e^−^ + 9H^+^ → NH_3_ + 3H_2_O), which necessitates effective activation and hydrogenation steps [8, 9, 10, 11]. Thus, the design of advanced electrocatalytic interfaces that promote rapid electron transfer and efficient proton availability is essential to achieving high ammonia production rates and superior faradaic efficiency [12, 13, 14, 15, 16, 17, 18, 19].

Covalent organic frameworks (COFs) are crystalline, porous polymers constructed from light elements (C, H, B, N, O) via dynamic covalent bonds. Since their initial discovery in 2005, COFs have garnered significant attention due to their high surface areas, tunable porosity, and ordered π‐conjugated backbones, making them suitable for applications in gas storage, separation, catalysis, sensing, energy storage, and optoelectronics [20, 21, 22, 23]. The ability to precisely incorporate functional groups into the framework endows COFs with the versatility required for heterogeneous catalysis and emerging electrochemical transformations [24, 25].

Recent advancements have explored the potential of COFs in electrocatalysis, particularly for the electrochemical nitrate reduction reaction (NO_3_RR) to ammonia, offering a sustainable alternative to the energy‐intensive Haber–Bosch process [26]. COFs provide a tunable platform to host catalytically active sites that promote selective NO_3_^−^ activation while suppressing competing hydrogen evolution reactions. For instance, Teng et al. introduced a bicopper COF (CuTAPc‐CuBpy‐COF) achieving a high NH_3_ Faradaic efficiency (FE) of 69.6% by integrating dual Cu sites for synergistic activation [27]. Similarly, Zhu and co‐workers showed that tailoring the morphology of 2D Cu‐bipyridine COF films significantly improved NO_3_RR kinetics and mass transport, resulting in an impressive FE of 92.7% [28]. Other studies utilizing Fe‐ and Ni‐COFs and multivariate COF coatings further highlight the flexibility of COF architectures in tailoring local environments and electronic structures for selective nitrate‐to‐ammonia conversion [26, 29, 30, 31, 32].

In contrast, fully metal‐free COFs for nitrate reduction remain largely unexplored. While COFs composed exclusively of light elements offer attractive sustainability and structural tunability, achieving efficient multi‐electron nitrate reduction without metal sites presents fundamental challenges. The reaction requires stabilization of key intermediates such as NO_2_, NO, and NH_x_ species, as well as effective control over proton‐coupled electron transfer steps. In purely organic frameworks, these processes must be mediated by polarized covalent bonds, heteroatom functionalities, and extended conjugation rather than metal‐centered redox chemistry.

Among metal‐free COFs, imine‐linked frameworks represent one of the most extensively studied classes due to their synthetic accessibility. However, their limited electrical conductivity and moderate bond polarization may restrict strong adsorption of nitrate reduction intermediates, and in the absence of engineered electrostatic bias, competitive hydrogen evolution can dominate under cathodic conditions [33, 34]. These considerations suggest that while conventional imine‐linked COFs provide a valuable platform, achieving high activity and selectivity in metal‐free NO_3_RR requires deliberate control of linkage chemistry and electronic structure.

Recent advances indicate that microenvironment regulation within COF pores can significantly influence nitrate reduction performance. For instance, incorporating cationic functional groups into COFs has been shown to generate localized positive electrostatic potentials that enrich nitrate anions while repelling protons, thereby suppressing hydrogen evolution and enhancing selectivity for NO_3_RR. Cheng et al. recently demonstrated this strategy using multivariate COF coatings, achieving a Faradaic efficiency of 91.0% for ammonia production in acidic media [26]. Such findings underscore that framework polarization and charge distribution are critical design parameters for metal‐free COF electrocatalysts.

In this context, ketazine‐linked COFs offer an attractive alternative linkage motif. The extended conjugation and enhanced electron delocalization associated with the ketazine unit can facilitate charge transport across the framework and potentially improve stabilization of reaction intermediates compared to simple imine linkages. In this work, we introduce a fluorine‐containing ketazine‐linked covalent organic framework (denoted F‐Ketazine COF) as a metal‐free electrocatalyst for NO_3_RR. Compared with the only previous report onX ketazine‐linked COFs by Jiang and co‐workers, a fluorinated building block is employed here, reducing the solvothermal synthesis time to 6 h while yielding a crystalline and porous material [35]. The incorporation of fluorine increases the electrophilicity of adjacent carbon centers and promotes more ordered π–π stacking interactions, thereby enhancing structural order. Moreover, the electron‐withdrawing nature of fluorine is expected to increase framework polarization and modulate the local electrostatic potential within the pores, potentially facilitating nitrate enrichment and influencing proton accessibility under electrochemical conditions.

For comparison, a non‐fluorinated ketazine COF (n‐Ketazine COF) was synthesized under modified conditions. Under neutral pH conditions, F‐Ketazine COF achieved a maximum Faradaic efficiency of 59.9% for NH_3_ at −0.9 V versus RHE, with an ammonia yield rate of 1639.9 µmol h^−^^1^ mgCOF^−^^1^. In contrast, n‐Ketazine COF reached a maximum Faradaic efficiency of 41% and a yield rate of 1113.4 µmol h^−^^1^ mgCOF^−^^1^ under identical conditions. To the best of our knowledge, this work represents the first application of a ketazine‐linked COF in electrocatalysis and the first demonstration of a metal‐free COF capable of electrochemical nitrate‐to‐ammonia conversion. More broadly, these results highlight the importance of linkage engineering and electronic modulation as key strategies for advancing metal‐free COF electrocatalysts in complex multi‐electron transformations.

## 2 Result and Discussion

### 2.1 Characterization

To investigate the importance of fluorine substitution and modulator use on COF crystallinity and formation kinetics, we first synthesized the fluorinated monomer 1,3,5‐trifluoro‐2,4,6‐tris(4‐acetylphenyl)benzene (TAB) [36]. This monomer was fully characterized by ^1^H and ^13^C NMR spectroscopy, as well as single‐crystal X‐ray diffraction, confirming its structure and purity (Figures S1–S3, Table S1). Fluorine atoms are known to enhance the electrophilicity of adjacent carbonyl groups and promote ordered π–π stacking interactions, which together facilitate the formation of highly crystalline frameworks [36, 37]. However, this elevated reactivity can also lead to rapid and uncontrolled polymerization, potentially suppressing long‐range order. To mitigate this, we introduced acetophenone as a modulator. By competing with TAB for the hydrazine linker, acetophenone reduces the effective concentration of the trifunctional monomer, thereby retarding the polymerization rate. This controlled slowdown prevents rapid precipitation and allows for the formation of a more thermodynamically stable, crystalline product [38, 39].

Using TAB and hydrazine, we synthesized a fluorinated ketazine‐linked COF (F‐Ketazine COF) via a solvothermal polycondensation reaction in the presence of trifluoroacetic acid (TFA) as a catalyst and a mixed solvent system of anisole and n‐butanol. The reaction was conducted at 120°C for varying durations (1, 2, 3, and 6 h; 1, 2, and 3 days). Among these, the sample synthesized after 6 h exhibited the highest crystallinity and the largest surface area, as determined by PXRD and N_2_ sorption, indicating that the combination of fluorine substituents and modulator use allowed for rapid but well‐controlled framework formation.

To further probe the roles of fluorine and the modulator, a non‐fluorinated analog of the ketazine COF was synthesized under identical conditions. In this case, no precipitation was observed when acetophenone was present, suggesting that in systems with intrinsically lower monomer reactivity, the modulator may overly suppress the condensation reaction. Upon modification of the reaction conditions, removing acetophenone, replacing TFA with acetic acid, and extending the reaction time to 5 days, we observed the emergence of weak crystalline features, confirming that prolonged reaction time and milder catalysis were necessary to achieve partial ordering in the absence of fluorine.

These findings collectively highlight the synergistic effects of fluorination and modulator choice in directing COF formation. Fluorinated monomers significantly increase reactivity and framework ordering through electronic and stacking effects but require modulators to control the reaction rate for optimal crystallinity. In contrast, non‐fluorinated systems benefit from slower, unmodulated conditions due to their inherently reduced reactivity. This study thus emphasizes the importance of tailoring reaction conditions to the electronic nature of the monomers in the rational design of crystalline COFs.

The chemical structure of the ketazine‐linked COFs was unambiguously confirmed by Fourier‐transform infrared (FT‐IR) spectroscopy, solid‐state ^13^C cross‐polarization magic angle spinning (CP/MAS) NMR, and ^19^F MAS NMR analyses. In the FT‐IR spectra, a prominent absorption band at 1607 cm^−1^ was assigned to the C═N stretching vibration, characteristic of the ketazine linkage present in both F‐Ketazine and n‐Ketazine COFs. For the fluorinated variant, an additional band at 1040 cm^−1^ was observed, corresponding to the C–F stretching vibration, thus indicating the presence of fluorinated moieties within the framework (Figure S4). The ^13^C CP/MAS NMR spectra further corroborated the formation of the ketazine linkage, exhibiting distinct signals at 128.1 and 126.4 ppm, which were assigned to the sp^2^‐hybridized carbon atoms of the C═N bonds. Moreover, signals at 10.18 and 12.13 ppm were attributed to the methyl groups in F‐Ketazine COF and n‐Ketazine COF, respectively, consistent with the expected chemical environments of the terminal acetyl units (Figure S5a,b).

Incorporation of fluorine atoms into the F‐Ketazine COF was confirmed by ^19^F MAS NMR spectroscopy. The spectrum displayed multiple well‐resolved resonances distributed over the range of −250 to 0 ppm, indicative of fluorine atoms residing in a range of chemically distinct local environments. These fluorine signals originate from the trifluorinated aromatic core of the 1,3,5‐trifluoro‐2,4,6‐tris(4‐acetylphenyl)benzene (TAB) linker, which is covalently integrated into the 2D COF architecture through ketazine condensation. The observed peak multiplicity and chemical shift dispersion reflect magnetic non‐equivalence among the three fluorine atoms of each TAB unit, likely arising from differences in electronic shielding induced by interlayer stacking, torsional distortions, and the anisotropic nature of the extended framework topology (Figure S6). While 19F–19F scalar coupling could in principle contribute, in many rigid extended aromatic solids (including COFs) the local environment effects (shielding, packing, CSA, etc.) are known to dominate the 19F line shapes [40, 41]. X‐ray photoelectron spectroscopy (XPS) was employed to further confirm the chemical composition and successful synthesis of F‐Ketazine COF and n‐Ketazine COF. In the case of F‐Ketazine COF, distinct signals corresponding to C 1s, N 1s, and F 1s were clearly observed, confirming the successful incorporation of fluorine atoms into the framework. In contrast, the XPS spectrum of n‐Ketazine COF showed only the characteristic C 1s and N 1s signals, with no detectable F 1s peak, verifying the absence of fluorine in this material. Notably, no peaks attributable to metals were detected in either spectrum, confirming that the frameworks are metal‐free within the detection limits of XPS and that the observed catalytic activity can be ascribed to the intrinsic framework rather than to residual metal species (Figures S7–S8).

**FIGURE 1:**
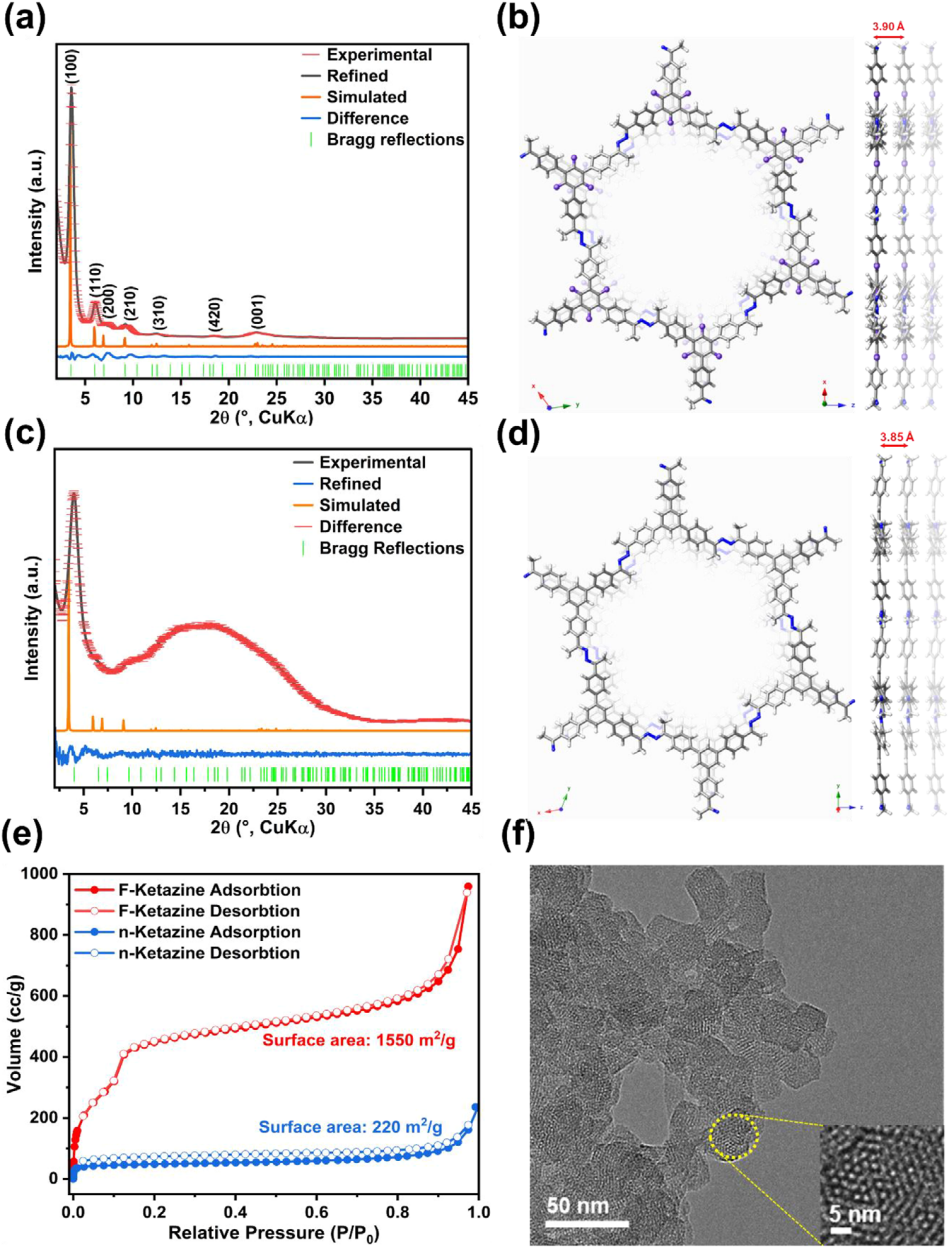
(a) Refined PXRD pattern of F‐Ketazine COF and assigned lattice planes with experimental pattern (red), pattern obtained after refinement (black), calculated pattern of simulated structure (orange), difference between refined and experimental pattern (blue), and Bragg reflection positions (green). (b) Simulated eclipsed **hcb** model of F‐Ketazine COF with fluorine substituents shown in purple, viewed along the (001) direction (left) and (100) direction (right), highlighting the layer distance. (c) Refined PXRD pattern of n‐ketazine COF with respective *R*_p_ and *R*_wp_ values. (d) Simulated structure of n‐Ketazine COF viewed from the (001) direction (left) and (100) direction (right) highlighting the interlayer distance. (e) N_2_ sorption isotherms of F‐ and n‐Ketazine COF at 77 K. (f) Low‐dose HRTEM images of F‐Ketazine COF.

The crystallinity of F‐Ketazine COF and n‐Ketazine COF was investigated by PXRD. Different solvothermal synthesis durations of F‐Ketazine COF were compared, showing the optimal time to be 6 h (Figure S9a,b). A structural model generated within the P6 space group (No. 186) based on eclipsed honeycomb (hcb) nets was found to be in best agreement with the experimental pattern (Figure S10). After Pawley refinement [42], a final model with a residual factor of *R*_p_ = 2.89% and a weighted residual factor of *R*_wp_ = 5.16% was obtained with final cell parameters of *a* = *b* = 29.577 Å, *c* = 3.909 Å and α = β = 90°, γ = 120°. The seven diffractions peaks at 2θ = 3.58°, 6.06°, 7.37°, 9.30°, 12.36°, 18.57°, and 22.86° were assigned to the (100), (110), (200), (210), (310), (420), and (001) lattice planes, respectively (Figure 1a). Notably, the (001) diffraction peak, corresponding to the interlayer stacking distance, appears at a smaller 2θ value compared to those typically reported for COFs. We attribute this to steric repulsion between the pendant phenyl rings of the TAB monomer, which adopt a twisted conformation, increasing the distance between adjacent layers.

In contrast to F‐Ketazine COF, n‐Ketazine COF displays only one diffraction peak at 2θ = 3.63°. As F‐ and n‐Ketazine COFs are isostructural, a simulated model was adapted from F‐Ketazine COF. Pawley refinement yielded a final model with a residual factor of *R*_p_ = 1.92% and a weighted residual factor of *R*_wp_ = 2.63% (final cell parameters: *P*6, No. 186, *a* = *b* = 29.613 Å, *c* = 3.851 Å, and α = β = 90°, γ = 120°) (Figure 1c). However, the PXRD pattern of the n‐Ketazine COF, which shows a broad (100) peak, nearly invisible higher‐order reflections, and a diffuse hump at higher angles, clearly indicates significantly lower crystallinity compared to its fluorinated analog. This suggests the presence of a substantial amorphous fraction. Although Pawley refinement yields low *R*_wp_ and *R*_p_ values, these parameters alone do not accurately reflect the agreement between the simulated and actual structure and should be interpreted carefully [43].

The porosity and surface areas of F‐Ketazine COF and n‐Ketazine COF were evaluated following vacuum activation to ensure complete removal of residual solvents. Nitrogen adsorption–desorption isotherms measured at 77 K confirmed that the pore networks in both materials were accessible to N_2_. The Brunauer–Emmett–Teller (BET) surface area of F‐Ketazine COF was calculated to be 1550 m^2^ g^−1^, significantly higher than that of n‐Ketazine COF, which exhibited a markedly lower value of 220 m^2^ g^−1^ (Figure 1e). Pore size distributions obtained via quenched solid density functional theory (QSDFT) revealed narrow and unimodal profiles. F‐Ketazine COF displayed a sharp maximum at 2.58 nm, in close agreement with the predicted pore diameter of 2.5 nm based on its structural model (Figure S11a). In contrast, n‐Ketazine COF showed a peak centered at 1.98 nm, noticeably deviating from the theoretical prediction. The lower crystallinity and diminished surface area of n‐Ketazine COF likely stem from weakened π–π interactions and enhanced structural disorder, which collectively hinder the formation of well‐defined, periodically aligned pores, resulting in restricted pore accessibility and an apparent reduction in effective pore size (Figure S11b). The morphology of F‐Ketazine and n‐Ketazine COFs was investigated using field emission scanning electron microscopy (FESEM), which revealed the formation of plate‐like structures composed of aggregated crystallites (Figure S12). The morphology and porosity of the F‐Ketazine COF were further investigated using high‐resolution transmission electron microscopy (HRTEM). As shown in (Figure 1f), the COF exhibits well‐defined nanoscale domains with clearly visible hexagonal pore arrays. The periodic contrast pattern across multiple particles confirms the high in‐plane crystallinity of the framework. These visible pores are in excellent agreement with the simulated structure and PXRD results, further supporting the formation of an ordered, porous 2D COF. In addition, energy‐dispersive X‐ray (EDX) analysis was also performed, confirming that no metal elements can be detected within the COF (Figure S13).

The thermal stability of F‐Ketazine‐COF and n‐Ketazine‐COF was assessed by thermogravimetric analysis (TGA) under a nitrogen atmosphere. Both materials exhibited thermal robustness, with no significant weight loss observed up to 350°C (Figure S14). Chemical stability tests were conducted by immersing the COF powders in 6 M HCl, 6 M NaOH, and boiling water for 7 days. Following exposure, the samples were thoroughly rinsed with deionized water, washed with acetone, dried, and subsequently analyzed by PXRD, which shows both COFs remained well‐defined after treatment with 6 M NaOH and boiling water, demonstrating their high structural stability under alkaline and hydrothermal conditions. In contrast, samples treated with 6 M HCl exhibited complete dissolution upon acetone washing prior to drying, with no recoverable solid material for PXRD analysis (Figure S15). This degradation is attributed to acid‐promoted hydrolysis of the ketazine linkage, where protonation of the C═N–N═C units facilitates cleavage into hydrazine and the corresponding ketone fragments, which are soluble in organic solvents such as acetone.

The efficacy of Pd removal, which was used during the monomer synthesis and may therefore have been introduced into the COF, was quantified by ICP‐OES and TXRF, revealing consistent residual palladium concentrations of only 0.003–0.004 wt% (31–38 ppm), respectively (Figure S16, Table S4). This value is significantly lower than the typical Pd loadings (∼1 wt%) employed in active Pd‐based catalysts for NO_3_RR [44, 45, 46]. Furthermore, the absence of Pd signatures in surface‐sensitive XPS and high‐resolution TEM/EDX conclusively excludes the presence of Pd nanoparticles or accessible catalytic sites within the detection limits of these techniques. These analyses collectively confirm that the COF is effectively metal‐free. Given the minimal Pd content, orders of magnitude below catalytically relevant loadings and undetectable by surface probes, the observed NO_3_RR activity cannot be attributed to residual palladium. This strongly suggests that the catalytic performance originates from the organic framework itself, pointing to a distinct, metal‐free mechanism for nitrate reduction.

### 2.2 Electrochemical Performance

All electrochemical experiments were performed using a CHI760E workstation at room temperature and ambient pressure using 0.1N KNO_3_ with 0.1N K_2_SO_4_ as an electrolyte solution in a three‐electrode H‐type electrochemical setup where an anodic chamber and a cathodic chamber are separated by a pre‐treated Nafion‐117 proton exchange membrane. A reference Ag/AgCl electrode and a catalyst loaded on carbon paper as a working electrode are placed in a cathodic chamber, and a counter electrode (Pt foil) was placed in an anodic chamber.

**FIGURE 2:**
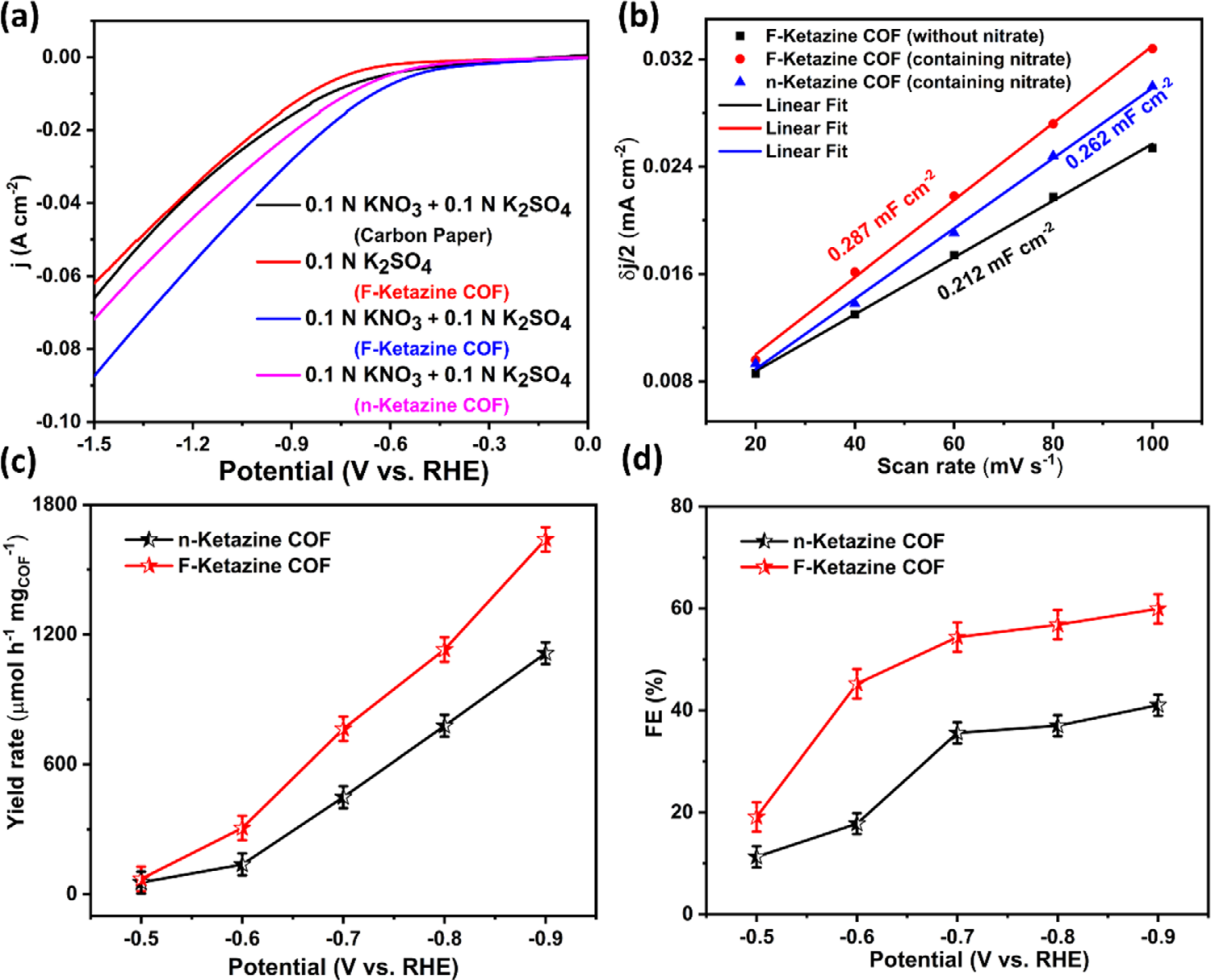
(a) LSV profile with and without nitrate‐containing electrolyte solution using bare carbon paper electrode, n‐Ketazine COF, and F‐Ketazine COF. (b) Double layer capacitance plot at various conditions using n‐Ketazine COF and F‐Ketazine COF. (c) Ammonia yield rate plot at various potential ranges using n‐Ketazine COF and F‐Ketazine COF. (d) FE at various potential ranges using n‐Ketazine COF and F‐Ketazine COF.

Linear sweep voltammetry (LSV) was used to investigate the NO_3_RR activity. As shown in Figure 2a, the electrolyte containing 0.1 N KNO_3_ and 0.1 N K_2_SO_4_ exhibits a higher current density compared to the nitrate‐free medium, confirming that nitrate reduction occurs under the applied conditions. In the absence of nitrate, the observed cathodic current originates from the hydrogen evolution reaction (HER) associated with water reduction. To decouple the contribution of the substrate, the bare carbon paper (CP) electrode was also examined in the nitrate‐containing electrolyte. The reduction current obtained was comparable to the HER background observed for the COF in nitrate‐free medium, indicating that bare carbon itself does not significantly catalyze NO_3_RR. In contrast, both COF‐modified electrodes display higher current densities in the presence of nitrate, with the F‐Ketazine COF showing the most pronounced enhancement. This demonstrates that the COFs actively promote NO_3_RR despite the absence of metal centers, which are typically regarded as the catalytic sites for this reaction. To the best of our knowledge, this is therefore the first metal‐free COF system active for NO_3_RR. To elucidate the role of surface charge in facilitating nitrate ion adsorption, the double‐layer capacitance (*C*_dl_) was evaluated from cyclic voltammetry (CV) measurements conducted in the non‐Faradaic region (Figure S17). As shown in Figure 2b, the F‐Ketazine COF exhibits a *C*_dl_ value of 0.212 mF cm^−2^ in the absence of nitrate ions. Upon nitrate addition, the *C*_dl_ value increases, indicating an expansion of the electrochemically active surface area. Notably, the F‐Ketazine COF achieves a higher *C*_dl_ of 0.287 mF cm^−2^, surpassing that of the n‐Ketazine COF (0.262 mF cm^−2^) (Tables S5–S6). From the *C*_dl_ values, we determined the electrochemical active surface area (ECSA); in this case, the ECSA value for F‐Ketazine COF was found to be 14.35 cm^2^, while that for n‐Ketazine COF was found to be 13.1 cm^2^. Figures S18–S20 and Table S7 also confirm that the charge transfer resistance (on electrode‐electrolyte surface) of F‐Ketazine COF is lower than that of n‐Ketazine COF, which indirectly indicates better NO_3_RR activity. We performed chronoamperometry (CA) experiments for 1 h at different potential windows (−0.5 to −0.9 V vs. RHE) to examine NO_3_RR activity for ammonia production using F‐Ketazine COF (Figure S21) and n‐Ketazine COF (Figure S22). After electrolysis for 1 h, the electrolyte was collected from the cathodic chamber for further ammonia detection (40 times diluted) using the indophenol blue method via UV‐vis spectroscopy.

Previously, a standard ammonia calibration series was developed using NH_4_Cl (NH_4_^+^ ↔ NH_3_ + H^+^) to measure unknown ammonia concentrations (Figures S23–S24). The maximum absorption peak for ammonia in the indophenol test reaches 656 nm. A similar absorption peak was observed for the electrolyte samples of both COF systems (Figures S25–S26), confirming the formation of ammonia during NO_3_RR. Figure 2c shows the ammonia production rates over different potential ranges for both COF systems. The highest ammonia production rate for F‐Ketazine COF was observed to be 1639.9 µmol h^−1^ mg_COF_^−1^ at −0.9 V versus RHE, while for the n‐Ketazine COF system, the value was is 1113.4 µmol h^−1^ mg_COF_^−1^. The highest Faradaic efficiency (FE) for F‐Ketazine COF was observed at –0.9 V versus RHE, reaching 59.9%, whereas n‐Ketazine COF achieved a maximum FE of 41% at the same potential (Figure 2d). As shown in Table S8, the ammonia production of F‐Ketazine COF is comparable to those of metal catalyst‐based COFs and carbons reported. We also evaluated the stability of the catalyst, and the F‐Ketazine COF system exhibited negligible change in current density during a 24‐h test (Figure S27). Moreover, the stability of the COF under reaction conditions was analyzed by various characterization methods. While PXRD analysis showed less intense diffraction peaks, pointing to a slightly reduced crystallinity (Figure S28), microscopy techniques (TEM and SEM) revealed no significant change in particle morphology after the 24‐h test (Figures S29–S30). This evidence for structural integrity is further corroborated by XPS. The high‐resolution N 1s and F 1s spectra exhibited only negligible shifts (≤ 0.2 eV) after the reaction, with peaks corresponding to characteristic nitrogen bonding environments (∼399 and ∼402 eV) and covalent C–F bonds (∼687 eV) remaining clearly resolved (Figure S31a–c). Additionally, post‐electrolysis XPS analysis showed no detectable Pd 3d signal (Figure S31d), confirming that trace residual Pd does not leach or redeposit on the surface during the reaction. This indicates that the local chemical states and bonding configurations of the framework's key heteroatoms are maintained, confirming the chemical robustness of the linkages and functional groups alongside the observed structural and morphological stability. We compared the ammonia results using the indophenol blue method with a UV‐Vis instrument with an ion chromatography (IC) instrument. The results show that there is no significant difference in the ammonia yield rate and Faradaic efficiency in the comparative analysis between the IC and UV‐Vis methods (Figures S32–S33). We calculated the mass activity of the electrocatalyst after electrolysis at a potential of −0.9 V versus RHE. The obtained mass activities are 306 and 266 A cm^−2^ g^−1^ for the F‐Ketazine COF and n‐Ketazine COF systems, respectively (Figure S34). Similarly, turnover frequency calculated and maximum TOF (s^−1^) observed at ‐0.9 V versus RHE for the F‐Ketazine COF and n‐Ketazine COF systems were 0.25 and 0.21 s^−1^, respectively (Figure S35). To decouple surface area effects from intrinsic catalytic activity, the NO_3_RR performance was further normalized using electrochemically relevant metrics, including double‐layer capacitance and turnover frequency; a comparative summary of BET surface area, *C*_dl_, mass activity, and TOF is provided in Table S9.

**FIGURE 3:**
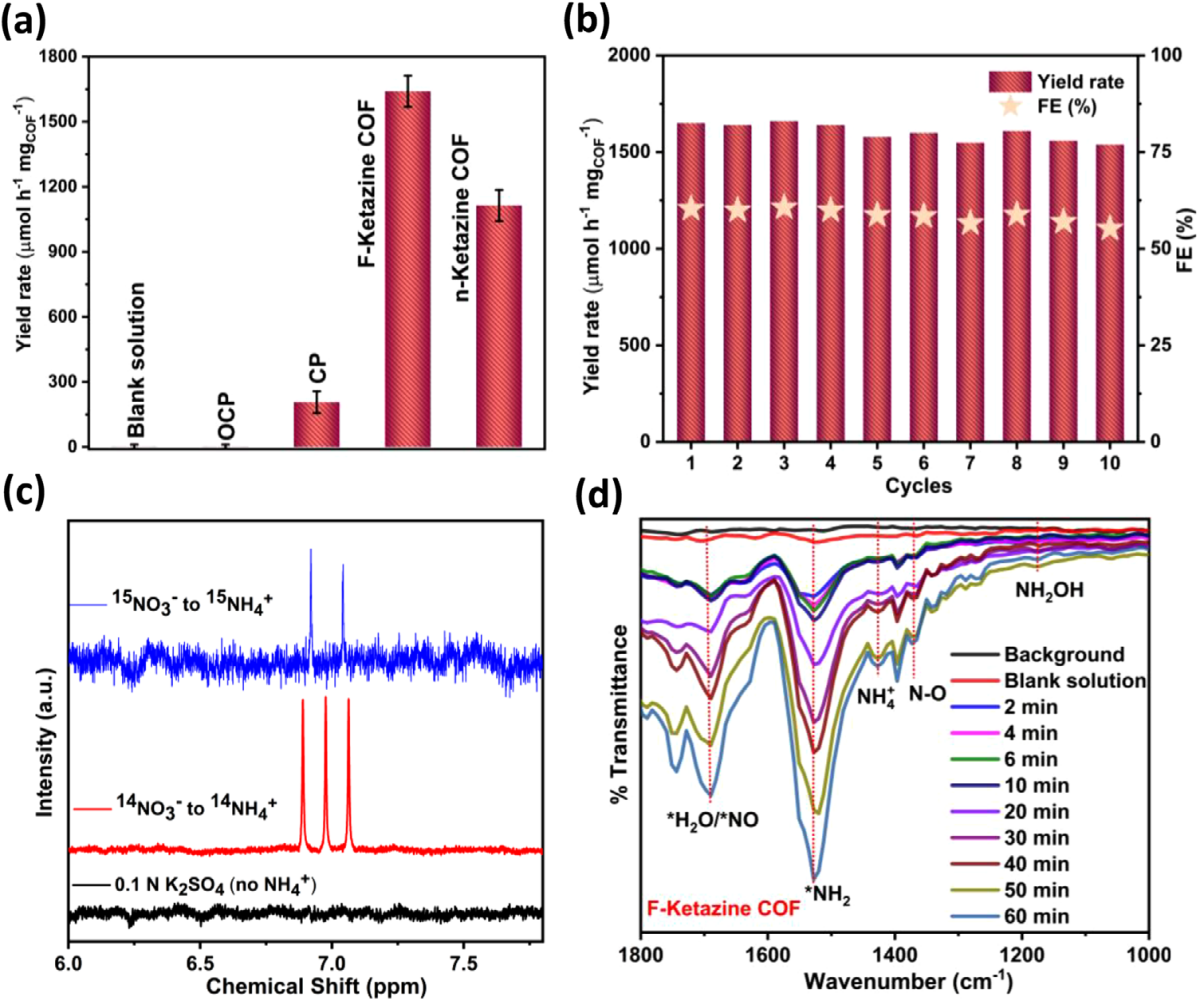
(a) Control experiments under various conditions. (b) Yield rates of NH_3_ and FEs for NH_3_ formation using F‐Ketazine COF for 10 cycles. (c) ^1^H‐NMR measurement after NO_3_RR using isotope‐labeled nitrate. (d) In situ ATR‑FTIR spectra of F‑Ketazine COF during NO_3_RR. The blank spectrum (red) was in 0.1 N K_2_SO_4_ without nitrate.

To examine the origin of ammonia, we conducted control experiments and isotopic experiments. The control experiment confirmed that the amount of ammonia was negligible in the sulfate electrolyte solution (without nitrate) and when the experiment was conducted under open circuit potential (OCP) conditions. A small amount of ammonia was detected when using CP as a catalyst in the nitrate‐containing 0.1 N K_2_SO_4_ electrolyte. However, significant amounts of ammonia were observed in both COF systems in the nitrate‐containing 0.1 N K_2_SO_4_ electrolyte solution (Figure 3a). Further, after 10 cycling tests, both NH_3_ yields and FEs show that the catalysts exhibit constant activity (Figure 3b and Figure S36). Furthermore, the formation of nitrite as a by‐product was tested by measuring the yield rate and the FE activity after electrolysis. The obtained results are significantly lower compared to the results for ammonia. The highest nitrite yield rate for F‐Ketazine COF was observed to be 106.3 µmol h^−1^ mg_COF_^−1^ at −0.7 V versus RHE, and the highest FE of F‐Ketazine COF was observed to be 4.9% at −0.5 V versus RHE (Figure S37). Furthermore, the effect of electrolyte pH on the NO_3_RR reaction was investigated. To test NO_3_RR activity at different pH levels, we used 0.1 N H_2_SO_4_ (acidic), 0.1 N K_2_SO_4_ (neutral), and 0.1 N KOH (basic) containing a 0.1 N KNO_3_ solution (Figure S38). In an acidic medium, ammonia production is low due to competitive HER reactions. In neutral and basic media, ammonia production is almost similar, but high pH can damage catalyst stability and reusability. We quantified the amount of H_2_ produced during electrolysis using gas chromatography (GC). The maximum amount of H_2_ produced at a potential of −0.9 V versus RHE for the F‐Ketazine COF and n‐Ketazine COF systems was 67.2 µmol h^−1^ mg_COF_^−1^ and 337.9 µmol h^−1^ mg_COF_^−1^, respectively (Figure S39).

Next, ^1^H‐NMR was performed at 600 MHz (Bruker) under acidic conditions (using 0.05 M H_2_SO_4_) with isotope‐labeled nitrate and DMSO‐d_6_ as the solvent. Figure 3c shows a signature triplet of ^14^NH_4_^+^ peaks at 6.889, 6.976, and 7.063 ppm (with two adjacent peaks splitting: ∼52 Hz) when the experiment was run using ^14^NO_3_^−^ as the electrolyte. Similarly, signature doublet in the ^1^H NMR spectrum corresponding to ^15^NH_4_^+^ at 6.920 and 7.042 ppm (with two adjacent peaks splitting: ∼73 Hz) was observed when the experiment was run using ^15^NO_3_^−^ as the electrolyte. Crucially, control experiments conducted in a sulfate‐only electrolyte, in the absence of nitrate, yielded no detectable ammonia. This confirms that the produced NH_3_ originates exclusively from the electrochemical reduction of nitrate ions, rather than from other nitrogen sources such as the catalyst framework. Furthermore, Figure 3d shows the in‐situ ATR‐FTIR spectrum during the NO_3_RR process. No FTIR vibrational peaks were observed in the blank solution (0.1 N K_2_SO_4_ electrolyte solution). The peak at ∼1691 cm^−1^ can be assigned to the formation of an NO intermediate or vibration band of adsorbed H_2_O [16] while the peaks at ∼1428 and ∼1539 cm^−1^ can be assigned to a ‐NH_2_ intermediate [47, 48]. The peak at ∼1370 cm^−1^ corresponds to the stretching vibration mode of the N–O bond of nitrate [47, 49]. The peak at ∼1176 cm^−1^ corresponds to a NH_2_OH species [28, 50]. These spectra help in understanding the reaction pathway (NO_3_^−^→ NO → *NH_2_ → NH_3_), which is also supported by DFT analysis.

### 2.3 Theoretical Studies

**FIGURE 4:**
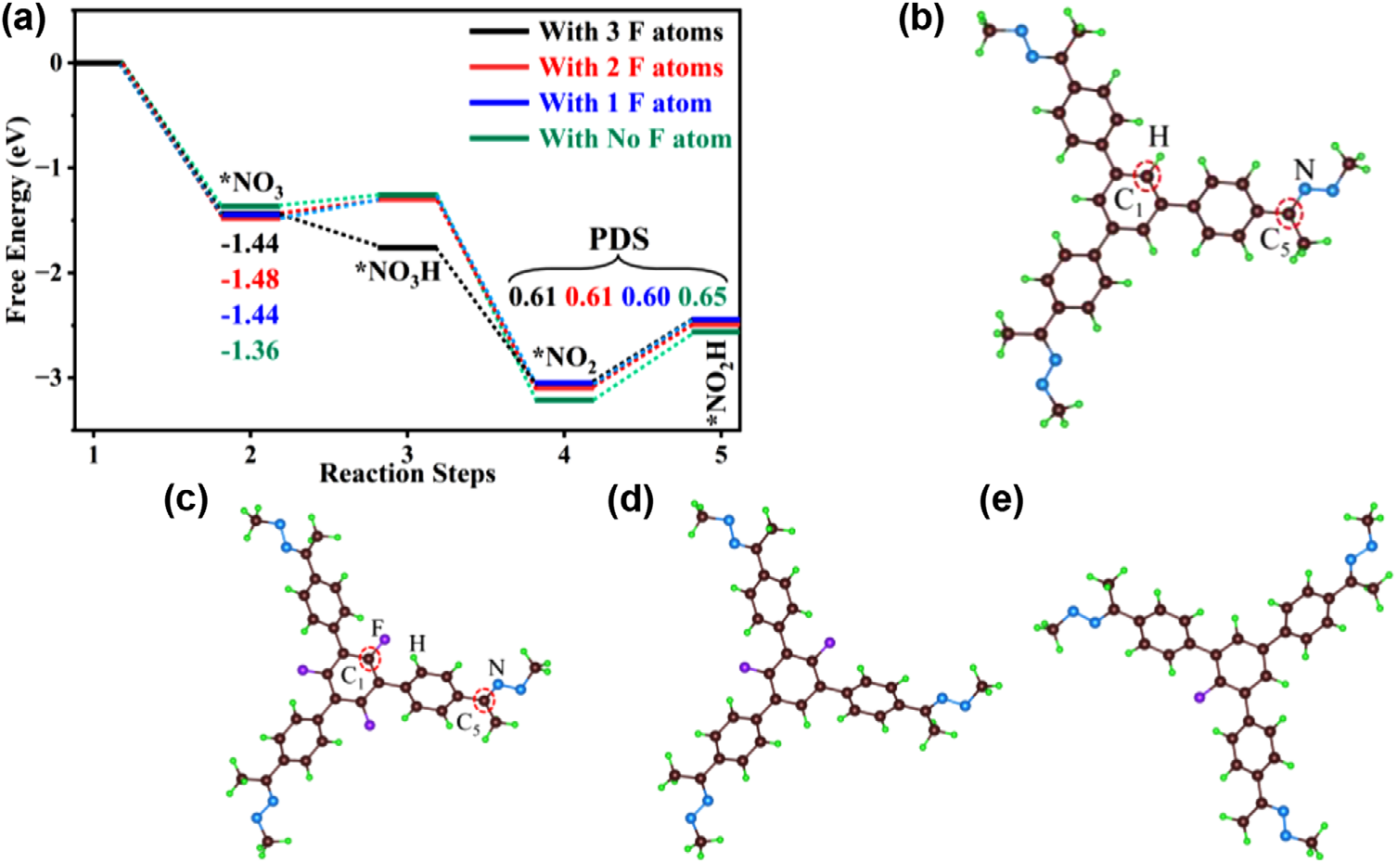
(a) Comparison of adsorption free energies for key intermediates (*NO_3_, *NO_2_, *NO_2_H) and limiting potentials for F‐Ketazine with three, two, and one fluorine atoms, and n‐Ketazine without fluorine. (b–e) Optimized structures of (b) n‐Ketazine and F‐Ketazine with (c) three, (d) two, and (e) one fluorine substituent.

Theoretical investigations were performed on representative repeating units of F‐Ketazine and n‐Ketazine COFs to elucidate the origin of their catalytic performance (Figure 4). The reaction free energy profiles were constructed by evaluating the adsorption energies of key nitrate reduction intermediates up to *NO_2_H and determining the corresponding limiting potentials. Four systems were considered: F‐Ketazine with three, two, and one fluorine substituents, and n‐Ketazine COF.

To quantitatively assess the influence of fluorine substitution, we calculated NO_3_^−^ binding energies and limiting potentials for all four systems (Table S10). Across the fluorinated models, NO_3_^−^ adsorption energies are comparable (−1.44, −1.48, and −1.44 eV for three, two, and one F atoms, respectively) and slightly stronger than that of n‐Ketazine (−1.36 eV), indicating that fluorination enhances reactant activation. However, the degree of fluorination (1–3 F atoms) does not significantly affect the binding energy, suggesting that the presence of fluorine, rather than the exact number of substituents, is the primary factor modulating the electronic environment.

The calculated potential‐determining step (PDS) is the hydrogenation of *NO_2_ to *NO_2_H, with limiting potentials of 0.61, 0.61, and 0.60 eV for F‐Ketazine with three, two, and one F atoms, respectively, compared to 0.65 eV for n‐Ketazine. The small difference between fluorinated and non‐fluorinated systems (0.04–0.05 eV) lies within the typical accuracy of DFT calculations and therefore does not represent a decisive energetic distinction.

Importantly, for the fully fluorinated system (three F atoms), the initial hydrogenation step (*NO_3_ → *NO_3_H) is exothermic, in contrast to the other three cases where this step is endothermic. This thermodynamically favorable early protonation step stabilizes key intermediates and creates a smoother overall free energy landscape. Taken together, the slightly stronger NO_3_^−^ adsorption and the favorable early‐stage hydrogenation in F‐Ketazine provide a mechanistic rationale for its superior experimental performance, despite the comparable limiting potentials [51]. To ensure the structural integrity of the catalyst models under operating conditions, we performed ab‐initio molecular dynamics (AIMD) simulations. The stability of the host framework is crucial, as the subsequent free energy profiles for the reaction are calculated based on these optimized structures. AIMD simulations were carried out at 300 K for 5200 fs with a time step of 2 fs. As shown in Figure S40, the optimized structures of both ketazine catalysts remain intact throughout the simulation, with no bond breaking or major distortion observed, and the total energy fluctuates around a stable equilibrium. This confirms that the employed catalyst models are thermally robust at room temperature, providing a reliable foundation for the mechanistic study.

**FIGURE 5:**
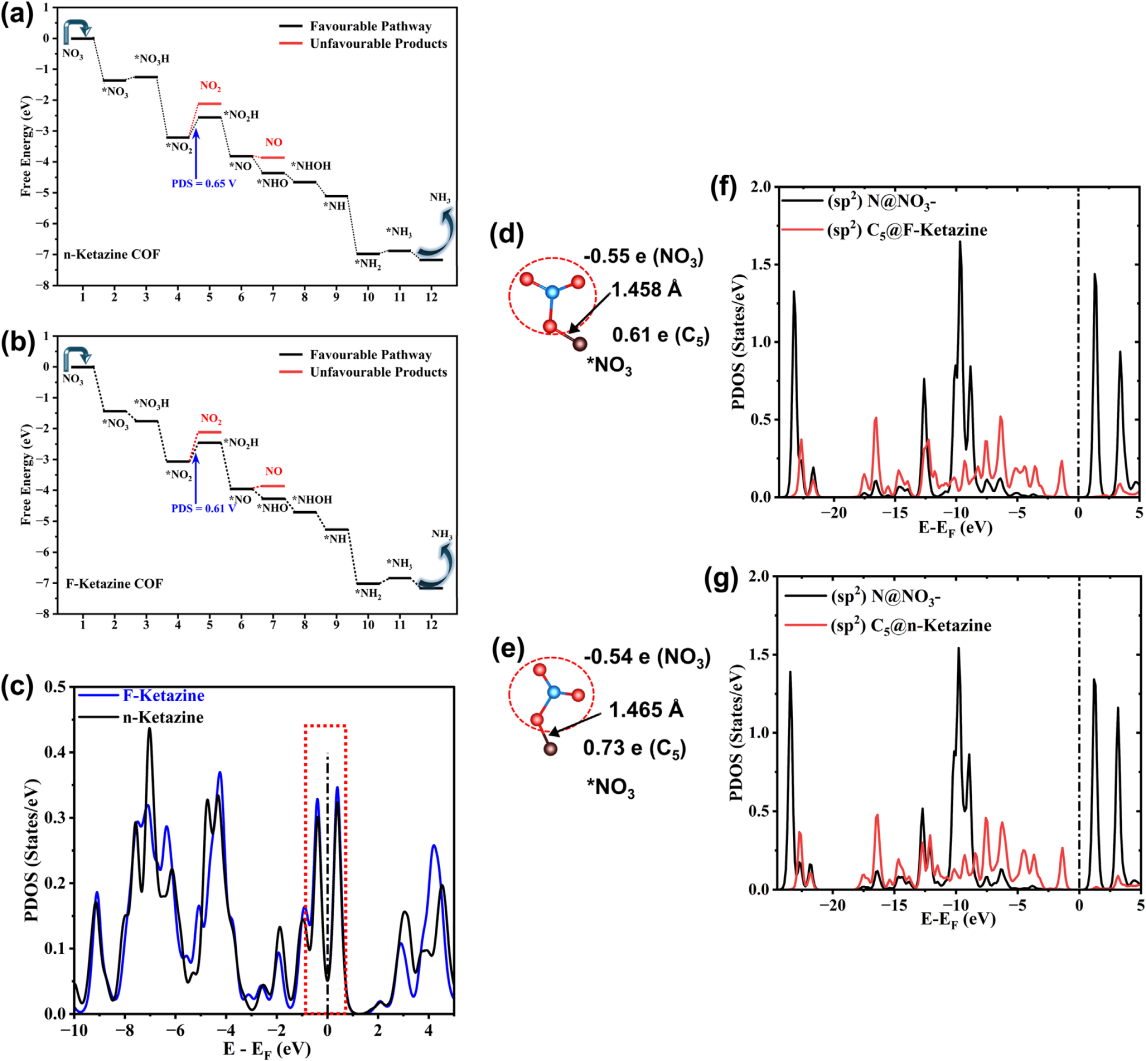
(a) and (b) Free energy profiles for n‐ketazine and F‐ketazine COF, (c) plot of PDOS of active site with rectangular box inset, (d) and (e) optimized adsorbed NO_3_ molecule, and (f) and (g) coupling of sp^2^ hybridized orbital of adsorbed NO_3_^−^ with sp^2^ hybrid orbital of C_5_ site for F‐Ketazine and n‐ketazine COF.

The catalytic reactivity was evaluated using the free‐energy profiles shown in Figure 5a,b. As NO_3_RR is an eight‐electron transfer process, identifying the potential‐determining step (PDS) is critical, as it governs the overall reaction kinetics. During the reduction pathway, the NO_3_^−^ molecule initially approaches the catalyst surface and binds at the active site with adsorption free energies of −1.36 and −1.44 eV for n‐Ketazine and F‐Ketazine, respectively. Subsequent stepwise protonation starts taking place, ultimately leading to the formation and release of NH_3_ from the catalyst surface. Throughout the reaction pathway, the transformation from *NO_2_ to *NO_2_H was identified as the PDS, as it exhibits the highest uphill free‐energy change of 0.61 eV for n‐Ketazine and 0.65 eV for F‐Ketazine (Figure 5a,b) [12]. Notably, NH_3_ desorption is barrier‐free for both catalysts, indicating that once NH_3_ is formed, it desorbs spontaneously from the surface.

To assess selectivity toward NH_3_, the formation of possible side products such as NO_2_ and NO was examined by comparing their desorption barriers with the formation energies of the corresponding intermediates (*NO_2_H and *NHO). In both cases, the side‐product pathways exhibit higher thermodynamic barriers than the main reaction intermediates, suggesting effective suppression of NO_2_ and NO formation on both ketazine catalysts (Figure 5a,b). Since the hydrogen evolution reaction (HER) is often kinetically more favorable than the multistep NO_3_RR, the H‐atom adsorption free energy was computed to assess its competitive influence. The adsorption free energies of NO_3_^−^ on both n‐Ketazine and F‐Ketazine are significantly more favorable than that of H adsorption. Thus, although the limiting potentials of both catalysts are lower than that required for HER, the stronger NO_3_^−^ adsorption ensures the NO_3_RR feasibility over HER (Figure 42c, d).

Based on the binding energy and limiting potential analysis, F‐Ketazine exhibits superior catalytic activity compared to n‐Ketazine. This enhanced performance is further supported by electronic structure calculations, including projected density of states (PDOS) and Bader charge analysis of the active sites, as well as an evaluation of the NO_3_^−^‐active site bond strength. In the case of F‐Ketazine, a higher density of electronic states is observed near the Fermi level compared to n‐Ketazine, as highlighted by the red rectangular region in the PDOS plots (Figure 5c–e). Bader charge analysis reveals greater charge transfer to the adsorbed NO_3_^−^ on F‐Ketazine (−0.55 e) than on n‐Ketazine (−0.54 e). Consistently, the shorter N–O bond length of adsorbed NO_3_^−^ on F‐Ketazine (1.458 Å) compared to n‐Ketazine (1.465 Å) indicates stronger adsorption (Figure 5d,e). Collectively, these results confirm the enhanced NO_3_^−^ adsorption and higher catalytic activity of F‐etazine relative to n‐ketazine [12, 52]. We also provided charge density difference plots to visualize the charge transfer during NO_3_^−^ adsorption on the active sites for both ketazine catalysts (Figure S41a,b). The strong NO_3_^−^ binding, in both the ketazines can be demonstrated by the observed strong coupling of the orbitals between sp^2^ hybridized orbital of the adsorbed NO_3_^−^ and sp^2^ hybridized orbital of C_5_ site, which are depicted in Figure 5f,g), As a result, charge transfer occurs smoothly for the further reaction steps.

## 3 Conclusion

In summary, we report a metal‐free, fluorinated ketazine‐linked COF (F‐Ketazine COF) as a highly efficient electrocatalyst for NO_3_RR under neutral conditions. In the F‐Ketazine structure (compared to the n‐Ketazine structure), the fluorine atoms withdraw electron density from the COF system, which strengthens π–π stacking interactions and facilitates linkage formation, leading to higher crystallinity and enhanced NO_3_RR performance. The COF was successfully synthesized within only 6 h, yielding particles with an average size of ∼40 nm and exhibiting high crystallinity, as confirmed by structural analysis. F‐Ketazine COF achieves an ammonia FE of 59.9% with a production rate of 1639.9 µmol h^−1^ mg_COF_^−1^ at −0.9 V versus RHE under ambient conditions. This work demonstrates that ketazine linkages are effective for designing stable, metal‐free COF electrocatalysts, paving the way for future green applications.

## Author Contributions

**Islam E. Khalil**: conceptualization, investigation, writing – original draft, validation, visualization. **Ashadul Adalder**: conceptualization, investigation, methodology, validation. **Badr Elkamash**: conceptualization, investigation, methodology, validation. **Narad Barman**: investigation, methodology. **Darosch Asgari**: investigation, methodology. **Luoxing Xiang**: investigation, methodology, validation. **Warisha Tahir**: investigation, methodology, validation. **Franziska Hess**: investigation, supervision, project administration, resources, writing – review and editing. **Ranjit Thapa**: investigation, methodology, validation. **Adisak Boonchun**: investigation, methodology. **Uttam Kumar Ghorai**: conceptualization, investigation, funding acquisition, writing – review and editing, methodology, supervision, project administration, resources. **Prasenjit Das**: investigation, supervision, validation, methodology, writing – original draft. **Arne Thomas**: conceptualization, funding acquisition, writing – review and editing, project administration, supervision, resources.

## Conflicts of Interest

The authors declare no conflicts of interest.

## Supporting information




**Supporting File 1**: anie72918‐sup‐0001‐Data.zip.

**Supporting File 2**: anie72918‐sup‐0002‐SuppMat.pdf.

## Data Availability

The data that support the findings of this study are available from the corresponding author upon reasonable request.
