# Supplementary material for: Ketazine‐Linked Covalent Organic Framework for Metal‐Free Electrocatalytic Nitrate‐to‐Ammonia Conversion
Source: Angew Chem Int Ed Engl. 2026 Jun 3;65(31):e6570629. doi: 10.1002/anie.6570629 (PMC13411222; doi:10.1002/anie.6570629)
Supplement: Supplementary file 2 — Supporting File 2: anie72918‐sup‐0002‐SuppMat.pdf. [file ANIE-65-e6570629-s002.pdf]

Ketazine-Linked Covalent Organic Framework for Metal-Free Electrocatalytic Nitrate-to-Ammonia Conversion

Islam E. Khalil,[1]* Ashadul Adalder,[2] Badr Elkamash,[3,4] Narad Barman,[5] Darosch Asgari,[1] Luoxing Xiang,[6] Warisha Tahir,[1] Franziska Hess,[3] Ranjit Thapa,[5,7] Adisak Boonchun,[8] Uttam Kumar Ghorai,[2]* Prasenjit Das, [1,9]* Arne Thomas[1,10]*

(1) Department of Chemistry, Functional Materials Technische Universität Berlin 10623 Berlin, Germany.

(2) Department of Industrial Chemistry & Applied Chemistry, Swami Vivekananda Research Centre, Ramakrishna Mission Vidyamandira, Belur Math, Howrah 711202, India.

(3) Institute for Chemistry, Technical University Berlin, D-10623 Berlin, Germany.

(4) Department of Mathematics and Computer Science, Faculty of Science, Alexandria University, Egypt.

(5) Department of Physics, SRM University AP, Amaravati 522 240 Andhra Pradesh, India

(6) Centre for Computational and Integrative Sciences, SRM University – AP, Amaravati 522 240, Andhra Pradesh, India

(7) Department of Physics, Faculty of Science, Kasetsart University, Bangkok, 10900, Thailand

(8) Thomas Lord Department of Mechanical Engineering and Materials Science, Duke University, Durham, North Carolina 27705, United States

(9) Department of Chemistry, Indian Institute of Technology Ropar, SSB Block, Rupnagar, Punjab, 140001 (India)

(10) Department of Chemistry, Chair of Macromolecular Chemistry, Technische Universität München, Lichtenbergstr. 4, 85748 Garching

Section S1. Materials and Instrumentation.

Chemicals: KNO_3 (Merck), K_2SO_4 (Merck), Nafion solution (Sigma-Aldrich), NaOH (Merck), salicylic acid (Merck), trisodium citrate (Merck), NaOCl solution (Merck), sodium nitroprusside dihydrate solution (Loba Chemie), NH_4Cl (Merck), H_2SO_4 (Merck), H_2O_2 (Merck), DI H_2O (Merck, Milli-Q), 2-propanol (Merck), Toray carbon paper (Thermo Scientific Chemicals), DMSO-D^6 (Cambridge Isotope Laboratories), etc.

X-ray Powder Diffraction patterns was collected on a Bruker D8 Advance diffractometer in reflection geometry operating with a Cu K_α anode (λ = 1.54178 Å) operating at 40 kV and 40 mA. Samples were ground and mounted as loose powders onto a Si sample holder. PXRD patterns were collected from 2 to 60 2θ degrees with a step size of 0.02 degrees and an exposure time of 2 seconds per step.

Field Emission Scanning Electron Microscopy (FESEM) was measured on a ZEISS GeminiSEM500. All the COF materials were observed directly without gold coating in nanoVP mode.

Nitrogen Sorption Measurements were performed at 77 K using an Autosorb-iQ-MP from Quantachrome. Prior to the analysis the samples were dried and degassed at 100 °C for 12 h.

Fourier transform infrared spectroscopy (FTIR) analyses were carried on Varian 640IR spectrometer equipped with an ATR cell.

Solid-state diffuse reflectance Ultraviolet–visible spectroscopy (UV-vis) spectra of the COFS have been collected on Varian Cary 300 UV-Vis Spectrophotometer.

^{1}H NMR Spectra for the samples dissolved in suitable solvents were carried on Bruker Avance II 400.

^{13}C Solid state NMR (cross polarization magic-angle spinning (CP/MAS)) spectra were carried out on a Bruker Avance 400 MHz spectrometer operating at 100.6 MHz.

X-ray Photoelectron Spectroscopy (XPS) was measured on a K-Alpha™ + X-ray Photoelectron Spectrometer System (Thermo Scientific) with Hemispheric 180 ° dualfocus analyzer with 128-channel detector. The X-ray monochromator used micro focused Al-Kα radiation. For the measurement, the powder samples were pressed and loaded on carbon tape, then pasted onto the sample holder for measurement. The data was collected with an X-ray spot size of 400 μm, 20 scans for the survey, and 50 scans for the regions.

High Resolution Transmission Electron Microscopy (HRTEM) was measured on a JEOL G-ARM STEM (JEM ARM300F2). All the COF samples were prepared on a carbon grid after suspension in a EtOH-acetonitrile mixture.

Thermogravimetric Analyses (TGA) were performed using a TGA Q500 thermal analysis system under a N_2 atmosphere from room temperature to 800 °C at a ramping rate of 1 °C /min.

ICP-OES quantification, 10 mg of F-Ketazine COF was digested in 4 mL of aqua regia at 150 °C overnight. Subsequently, a 330 μL aliquot of the resulting digest was diluted with 2.5 mL of deionized water prior to analysis.[1]

Total reflection X-ray fluorescence spectroscopy

The residual palladium content in the F-Ketazine COF was quantified by total reflection X-ray fluorescence (TXRF) spectroscopy. A 10 mg of the COF was digested in 4 mL of aqua regia at 150 °C overnight.

For measurement, a 2.5 μL aliquot of the digest was mixed with 2.5 μL of a Certipur yttrium internal standard solution (50 mg L^{-1}, Merck), resulting in a 1:1 dilution. A 5 μL aliquot of this mixture was deposited onto a siliconized quartz glass carrier (Bruker Nano) and dried on a hot plate at 50 °C.

Measurements were performed on a Bruker S4 T-Star spectrometer equipped with a tungsten anode X-ray source and an X-Flash silicon drift detector. The sample was excited for 600 s. A blank sample containing only aqua regia was prepared and measured identically to account for

any metal contamination from the reagents. The palladium concentration was determined relative to the yttrium internal standard using the Esprit 1.0 software package (Bruker Nano) for spectral analysis and deconvolution. Four technical replicates were measured per sample.

The residual Pd content in the COF was calculated after subtracting the concentration measured in the aqua regia blank (0.018 mg L^{-1}) from the concentration measured in the COF digest (0.066 mg L^{-1}). The final Pd content in the F-Ketazine COF was determined to be 0.0038 wt% (38 ppm) **(Figure S14)**.[2]

The working electrode (catalyst ink) was prepared by mixing 0.6 mg F-Ketazine COF or 1.1 mg n-Ketazine COF, 300 µL of 2-propanol and 20 µL of Nafion solution. The solution mixture was first sonicated for 2 min and then vortexed for 5 min to form a homogeneous mixture. Then, 80 µL (F-Ketazine COF)/40 µL (n-Ketazine COF) of the prepared mixture solution was drop-cast onto carbon paper (pre-treated with dilute H_2SO_4 and water), with a cross-sectional area of 1×1 cm^2 on both sides (mass loading of ~0.14-0.15 mg cm^{-2}) and dried for 4 h before use.

All the electrochemical performances were conducted with an Ag/AgCl reference electrode (saturated KCl). The found potentials were transformed to the reversible hydrogen electrode (RHE) scale with the help of the following equation:

$E_{RHE} = E_{Ag/AgCl} + 0.198\ V + (0.059\ x\ pH)$.

Where 0.198 V is the standard potential of Ag /AgCl (saturated KCl) electrode at 25 °C versus SHE.

A.C. impedance (Nyquist plot) was performed under initial E (V) = 0 & various potential, high frequency (Hz) = 1e+5, and low frequency (Hz) = 1.

The Nafion-117 membrane was pretreated with DI water at 80°C for 1 h, H_2SO_4 at 80°C for 1 h, 5% H_2O_2 solution for 1 h, and finally rinsed with DI water.

Ammonia/ammonium ions were detected after the NO_3RR process using a well-established indophenol blue method using UV-VIS spectroscopy (model: UV-3600 Plus). Before this, three essential stock solutions were prepared. Solution A was prepared by mixing 4 g NaOH (Merck), 5 g salicylic acid (Merck) and 5 g trisodium citrate (Merck) in 100 ml DI water. Solution B

contained NaOCl solution (Merck, 5 ml with 45 ml DI water). Solution C contained sodium nitroprusside dihydrate solution (Loba Chemie, 0.5 g in 50 ml DI water). Further, 2 ml of electrolyte solution (after NO_3RR) was mixed with 2 ml of solution A, 1 ml of solution B and 0.2 ml of solution C. The mixture solution was kept in a dark place for 2 hours and then a UV test was performed. A standard stock solution of ammonia (using NH_4Cl, Merck) was prepared for calculation purposes.

Equations:

$$\text{Ammonia yield rate} = \frac{(\mathrm{C \ x \ V})}{(\mathrm{m}_{\mathrm{cat.}} \ \mathrm{x \ t})}$$

$$\mathrm{FE}\ (\%) = \frac{(8 \ \mathrm{x \ F \ x \ C \ x \ V})}{(\mathrm{M \ x \ Q})} \ \mathrm{x} \quad 100\%$$

Where C is the concentration of the ammonia (μmol/ml, 40-time dilution), V is the volume of the electrolyte (ml), $m_{cat.}$ is catalyst (COF) weight (mg), t is the time of electrolysis (h), F is the faraday constant (96485 C/mol), M is the molar mass of ammonia, Q is the charge passing through the electrode surface.

We calculated the turnover frequency using the formula:

$$\mathrm{TOF} = \frac{(\mathrm{j \ X \ A})}{(8 \ \mathrm{nF})} \ (\mathrm{s}^{-1});\ \text{unit conversion} = \frac{\frac{A}{cm^2} \, x \, cm^2}{mol \, x \frac{C}{mol}} = \frac{A}{C} = \frac{A}{A \, x \ t} = \frac{1}{t}$$

In this equation, j (A cm^{-2}) is the current density measured at a specific potential. The parameter A represents the surface area of the working electrode. The number 8 is the electron transfer process for the reaction, which is important for the NO_3RR catalytic process. Here, n is the number of moles of catalyst used for the NO_3RR process. Finally, F is Faraday's constant, a fundamental constant in electrochemistry, with a value of 96485 C/mol.

Computational details

All the DFT simulations are performed using VASP computational code considering all spins polarized.[3–5] To demonstrate the external potential where core and valence electrons interact, PAW potentials were employed,[6] whereas electron-electron interactions managed with GGA PBE functional.[7] For the optimization of all the atomic structures, a conjugate gradient method was incorporated where ionic and electronic relaxations convergence criteria were set to be 10^{-04} and 10^{-06} for respectively. For structural optimization and electronic structure calculations, Gamma centre grid method was applied. To include long range Van der Waals (VDW) forces, D3-scheme of Grimme with zero damping force was incorporated.[8]

Identification of active site theoretically:

Theoretical investigations were carried out on two representative repeating units from F-Ketazine and n-Ketazine COFs (**Figure S41a, b**). Based on Bader charge analysis, the C5 site was selected for adsorption free-energy calculations due to its high induced charge, which promotes interaction with adsorbates. The site C1 was chosen for comparison, as its electronic environment is modified by the substituents (F in F-Ketazine and H in n-Ketazine) allowing us to contrast its binding energy for NO_3- with that of C5. Among them, the C_5 site was identified as the most favourable active site for the nitrate reduction reaction, as it exhibits significantly stronger NO_3- binding energies (-1.36 and 1.44 eV for n-Ketazine anf F-Ketazine respectively) compared to the C1 site (-0.52 and -0.60 eV for n-Ketazine anf F-Ketazine respectively) (**Figure S42c, d).**

Section S2. Synthesis of molecular building blocks and COFs.

Section S2.1 Synthesis of 1,3,5-trifluoro-2,4,6-tris(4-acetylphenyl)benzene (TAB):[9]

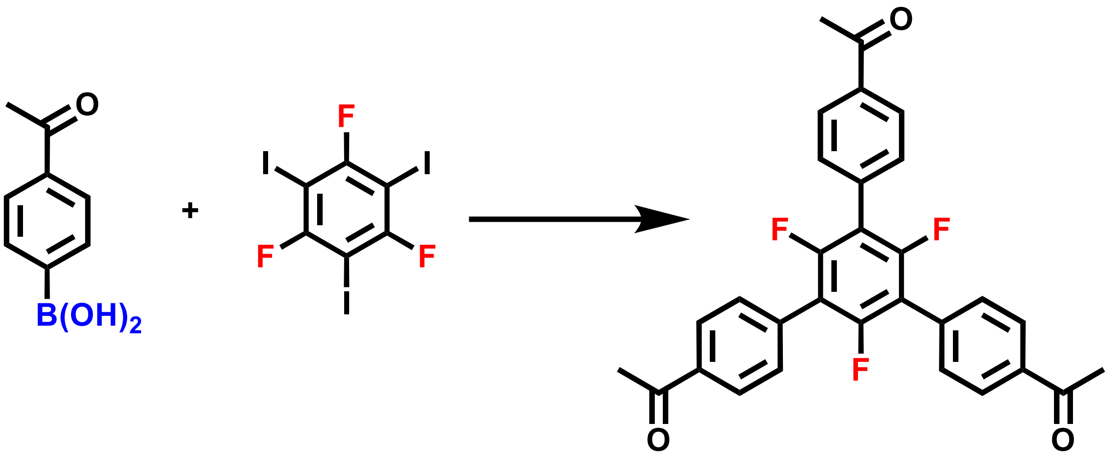

A mixture of 4-acetylphenylboronic acid (1.31 g, 8 mmol), 1,3,5-trifluoro-2,4,6-triiodobenzene (1.02 g, 2 mmol), K_2CO_3 (2.62 g, 19 mmol) and $Pd(PPh_3)_4$ (184 mg, 0.16 mmol) was charged in a 2-neck round bottom containing dioxane/water (100/30 mL). The mixture was purged with nitrogen for 15 minutes and heated under reflux overnight. After cooling to room temperature, the solvent was removed by a rotary evaporator. The crude product was extracted by DCM (50 mL * 3) and washed by water. The combined organic layers were dried by adding sodium sulfate. The product was purified by column chromatography using DCM/ethyl acetate (100:2) as eluent to obtain a white solid (853 mg, 88% yield). 1H NMR (400 MHz, DMSO) δ 8.11 – 8.09 (m, 6H), 7.77 (d, J = 8.16 Hz, 6H), 2.63 (s, 9H). ^{13}C NMR (400 MHz, DMSO) δ 197.56, 156.8, 136.74, 132.46, 130.71, 128.33, 114.23, 26.81.

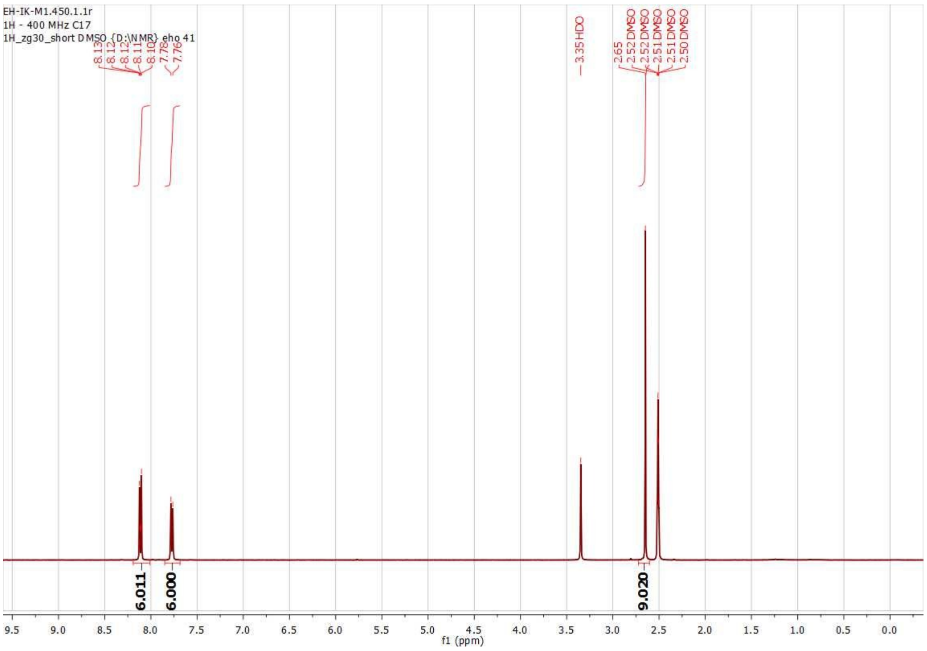

Figure S1. ^{1}H-NMR of 1,3,5-trifluoro-2,4,6-tris(4-acetylphenyl)benzene (TAB)

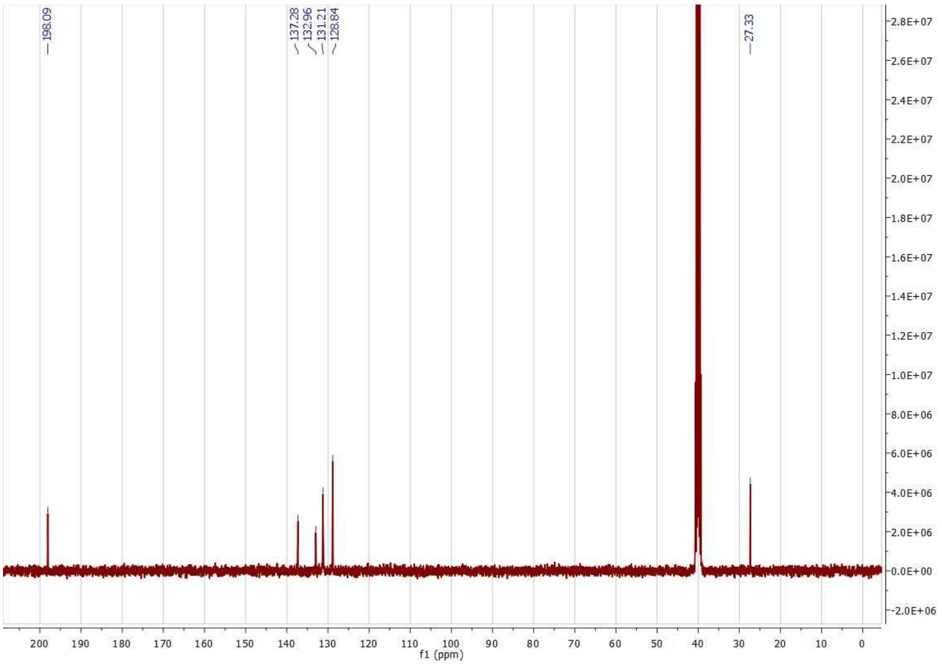

Figure S2. ^{13}C-NMR of 1,3,5-trifluoro-2,4,6-tris(4-acetylphenyl)benzene (TAB)

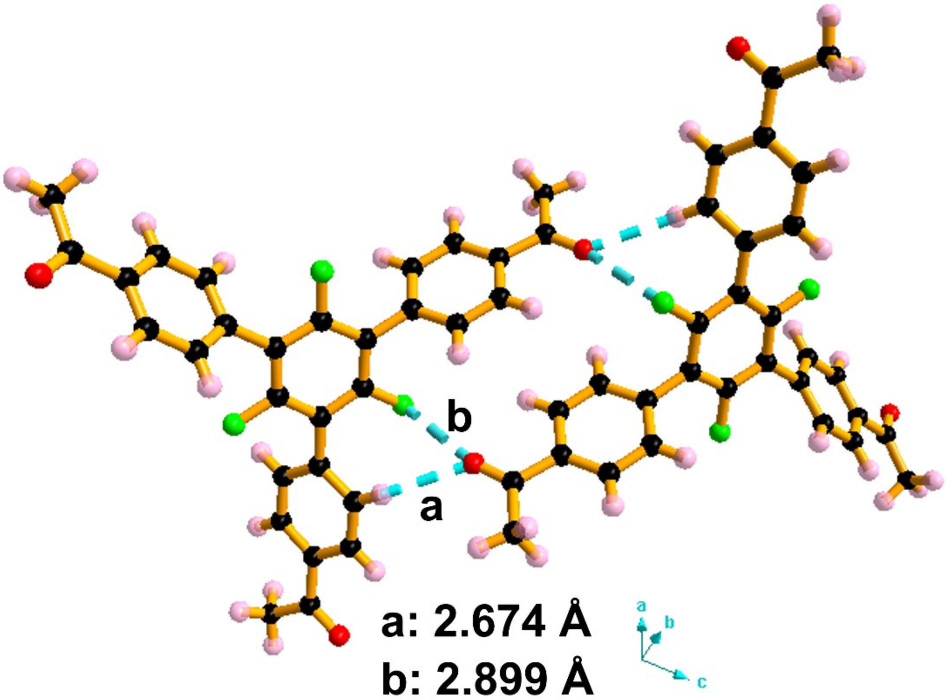

Figure S3. Crystal structure of the 1,3,5-trifluoro-2,4,6-tris(4-acetylphenyl)benzene (TAB) obtained from single-crystal X-ray diffraction (XRD) analysis. The molecular packing reveals intermolecular interactions (highlighted as turquoise dashed lines) with contact distances of a = 2.674 Å and b = 2.899 Å, indicative of short-range noncovalent interactions that may contribute to structural stability and molecular organization in the solid state.

Table S1. Crystallographic data and structure refinement parameters for the **Linker**.

compound	**Linker**
CCDC No.	2475337
chemical formula	$C_{30}H_{21}F_3O_3$
formula weight (g mol^{-1})	216.882
temperature (K)	150.00(10)
wavelength (Å)	0.71073
crystal system	monoclinic
space group	$P2_1$/c
a (Å)	12.2611(2)
b (Å)	7.53521(13)
c (Å)	25.6415(4)
α (°)	90
β (°)	102.0885(17)
γ (°)	90
Z	6
V (Å^3)	2316.50(7)
density (g/cm^3)	1.475
μ (mm^{-1})	0.883
F(000)	1011.724
2θ (°) range for data collection	3.53 to 72.60
no. of reflections collected	4463
no. of independent reflections	3811
no. of reflections with I >2σ(I))	9092

R_{int}	0.0201
no. of parameters refined	328
GOF on F^2	1.0524
final $R_1{}^a$ / w$R_2{}^b$ (I >2σ(I))	0.0380/ 0.0995
$R_1{}^a$ / w$R_2{}^b$ (all data)	0.0450/ 0.1064
largest diff. peak and hole (e Å^{-3})	0.3223/ -0.2111

$^aR_1 = \Sigma||F_o| - |F_c||/\Sigma|F_o|$. $^b\mathrm{w}R_2 = [\Sigma \mathrm{w}(F_o{}^2 - F_c{}^2)^2/\Sigma \mathrm{w}(F_o{}^2)^2]^{1/2}$, where

$\mathrm{w} = 1/[\sigma^2(F_o{}^2) + (aP)^2 + bP]$, $P = (F_o{}^2 + 2F_c{}^2)/3$.

Section S2.2 Synthesis of F-Ketazine COF:

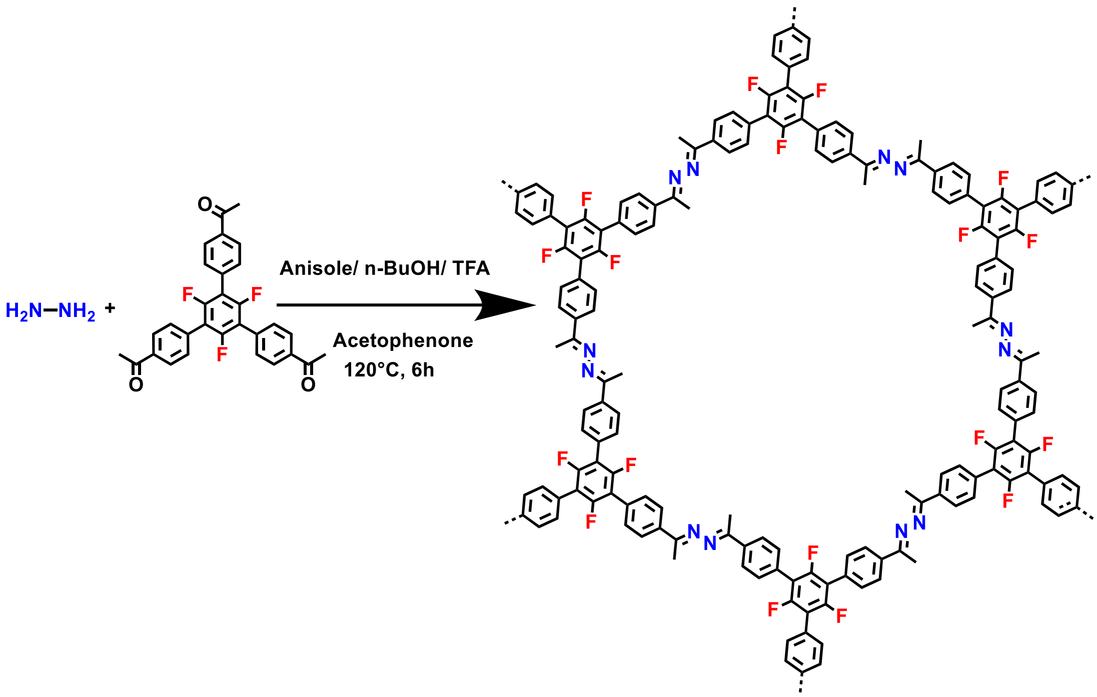

A 10 mL Pyrex tube was charged with TAB (47.6 mg, 98 μmol), hydrazine (7.1 μL, 45 μmol), and acetophenone (34.3 μL, 294 μmol) in a solvent mixture of anisole/n-butanol/pure TFA (75:25:6 vol%; total volume: 2.12 mL). The reaction mixture was degassed using three freeze–pump–thaw cycles, then the tube was sealed under vacuum and heated at 120 °C for 6h. After cooling to room temperature, the resulting precipitate was collected by suction filtration, washed thoroughly with THF (5×), and subsequently purified by Soxhlet extraction with THF overnight. The solid product was dried under vacuum at 120 °C overnight to yield the F-Ketazine COF as a powder in 70% isolated yield.

Section S2.3 Synthesis of n-Ketazine COF:

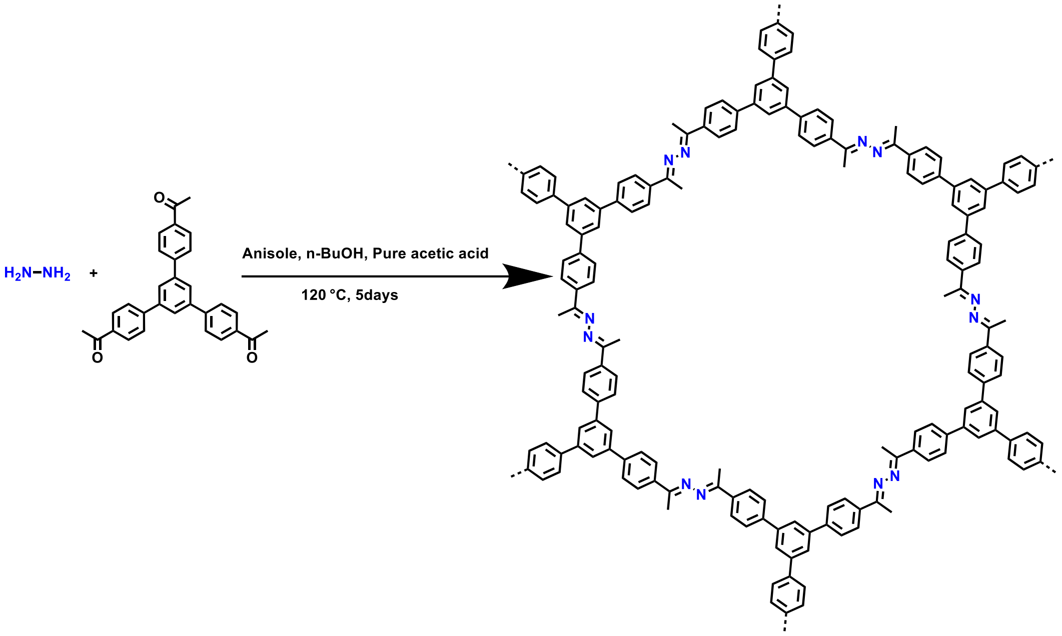

A Pyrex tube (10 mL) filled with 1,3,5-tri(4-acetylphenyl)benzene (25.9 mg, 60 µmol), hydrazine (7.1 µL, 45 µmol) in a mixture of anisole/n-BuOH/pure AcOH (80/20/6 vol.; 2.12 mL) was degassed by three freeze–pump–thaw cycles. The tube was sealed and heated at 120 °C for 5 days. The precipitate was collected by suction filtration, washed five times with THF, and extracted via Soxhlet with THF overnight. The powder was dried at 120 °C under vacuum overnight to afford n-Ketazine-COF in an isolated yield of 65%.

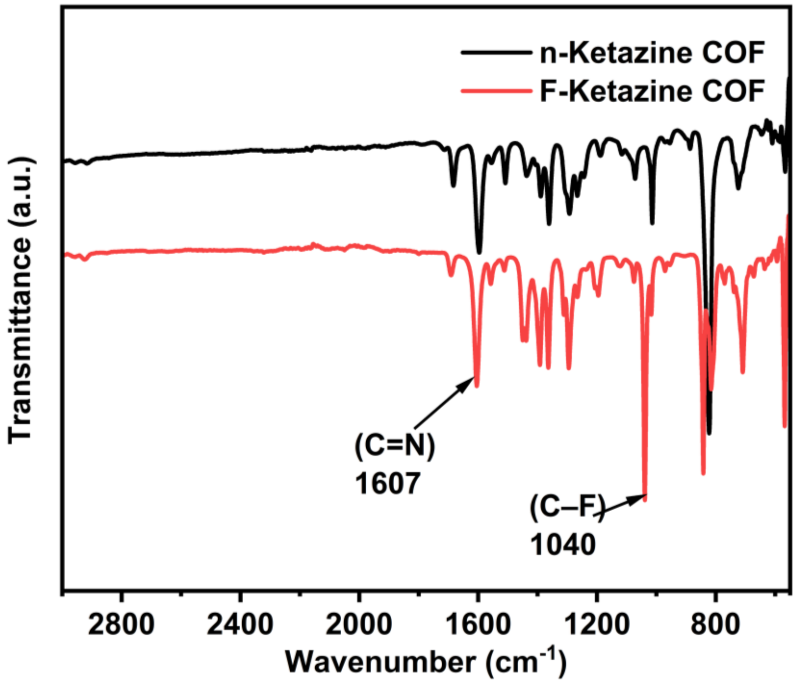

Figure S4. FT-IR spectra of F-Ketazine and n-Ketazine COFs

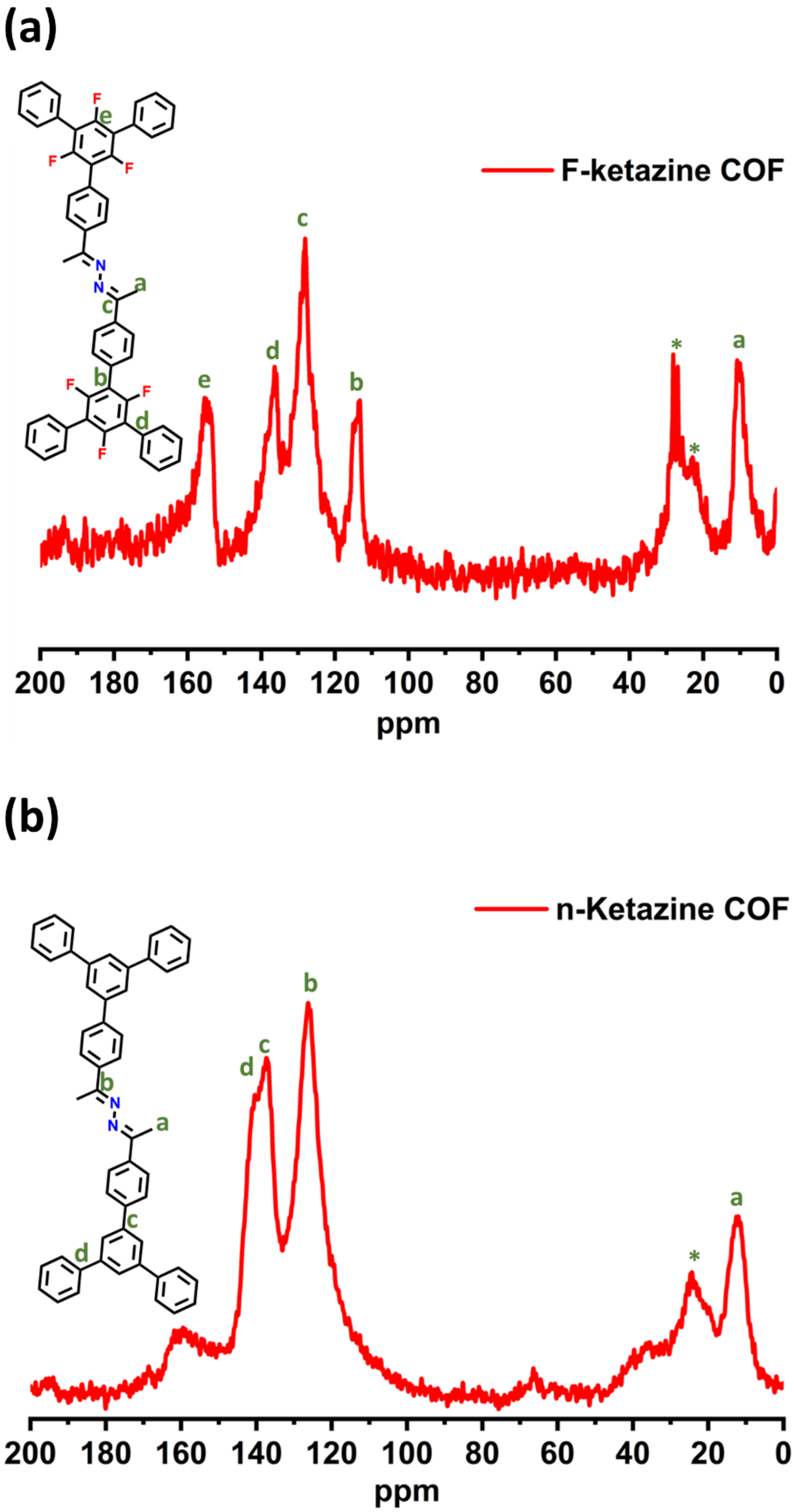

Figure S5. ^{13}C (CP/MAS) NMR spectrum of F-Ketazine, and n-Ketazine COFs

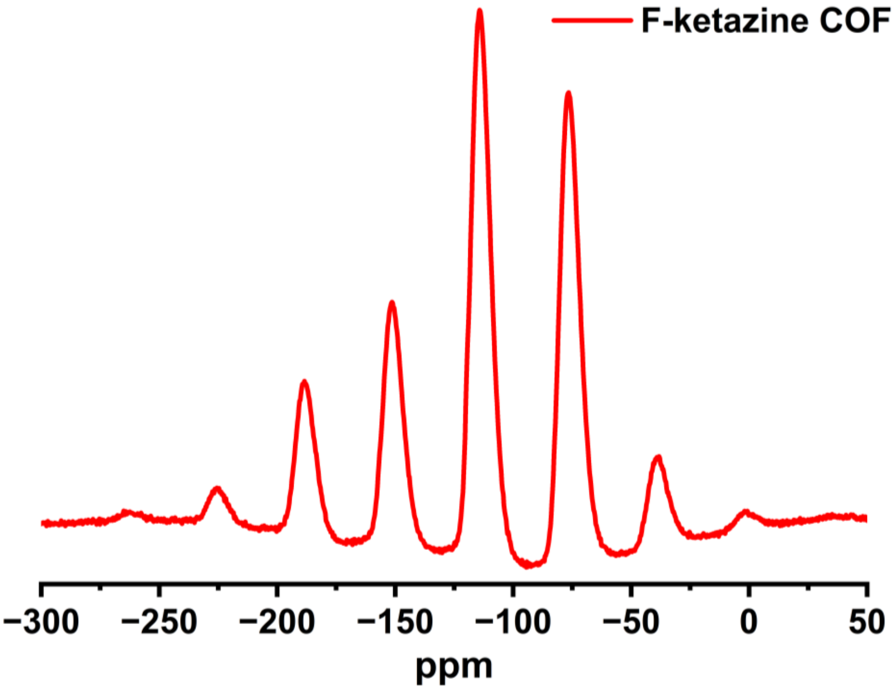

Figure S6. ^{19}F MAS NMR of F-Ketazine COF.

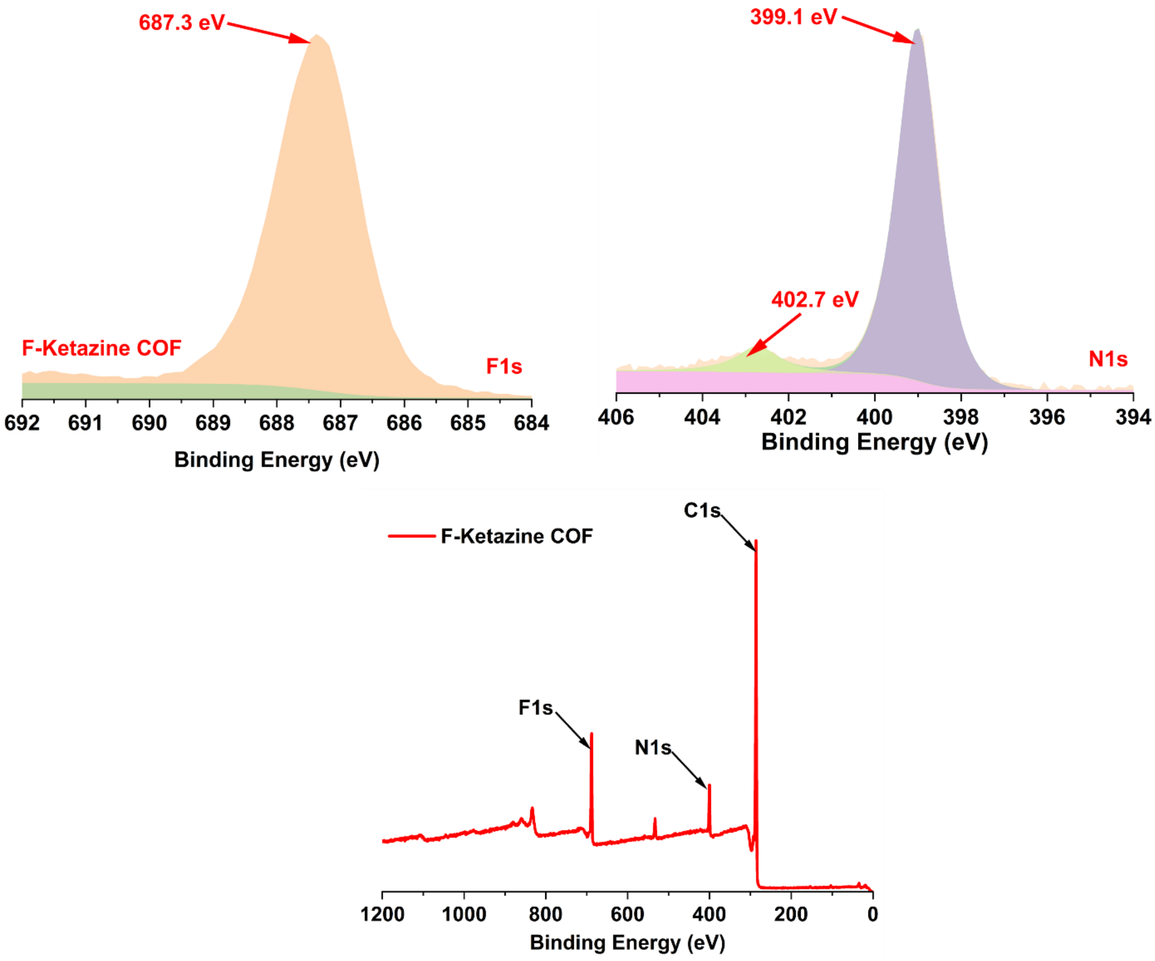

Figure S7. F 1s, N 1s and XPS survey spectra of F-Ketazine COF.

XPS survey scan with assigned peaks. Additional peaks can be attributed to the O1s photoelectron line at ~530 eV, and the corresponding OKLL and FKLL Auger peaks at ~975 eV and ~830 eV, respectively.[10,11]

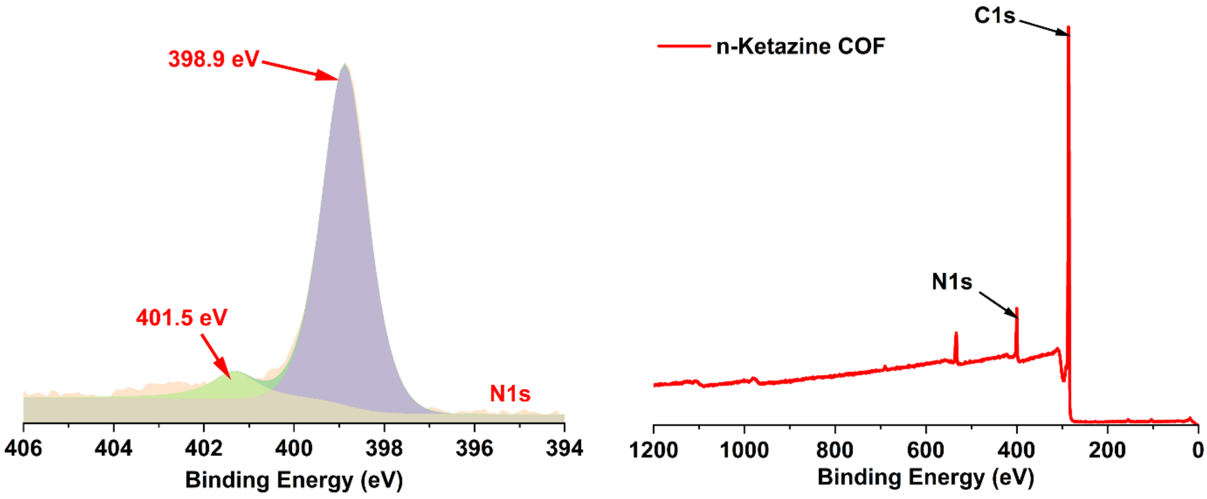

Figure S8. N1s and XPS survey spectra of n-Ketazine COF

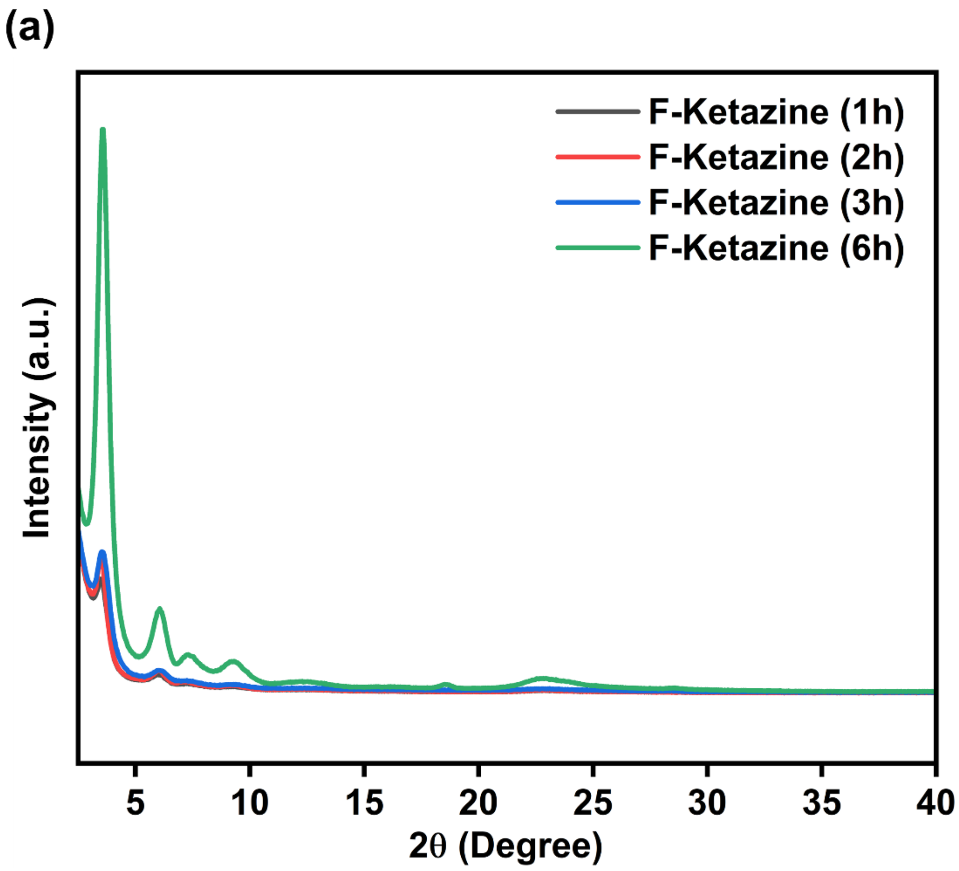

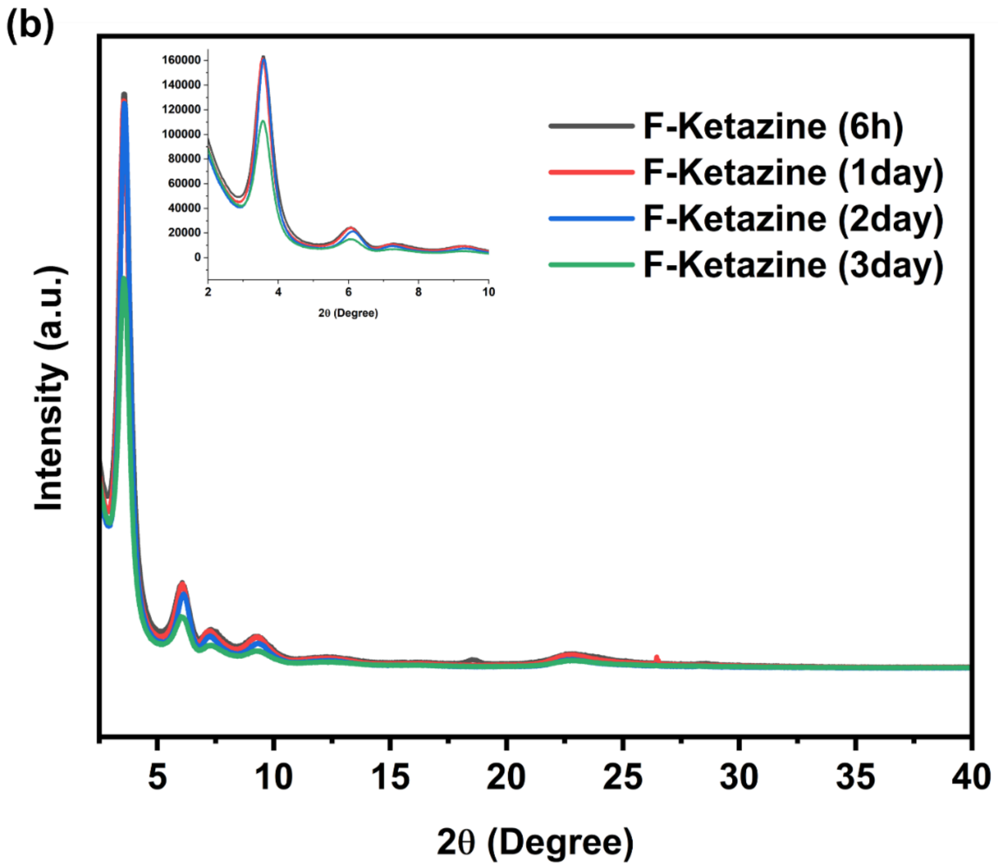

Figure S9. PXRD patterns of F-Ketazine COF synthesized at different reaction times (a) 1 h, 2h, 3 h, 6 h and (b) 1 d, 2 d, and 3 d reveal that the sample prepared at 6 h exhibits the highest crystallinity, as indicated by the sharpest and most intense diffraction peaks. Crystallinity is already evident at shorter times (1-3 h), though less pronounced. Notably, extending the reaction time beyond 6 h results in decreased peak intensity, suggesting a reduction in long-range order. This trend implies that 6 h represents the optimal duration for framework formation and ordering, likely due to a balance between monomer diffusion and dynamic bond reversibility that promotes error correction. In contrast, longer reaction times may lead to over-condensation, structural rearrangements, or side reactions that compromise the COF's crystallinity.

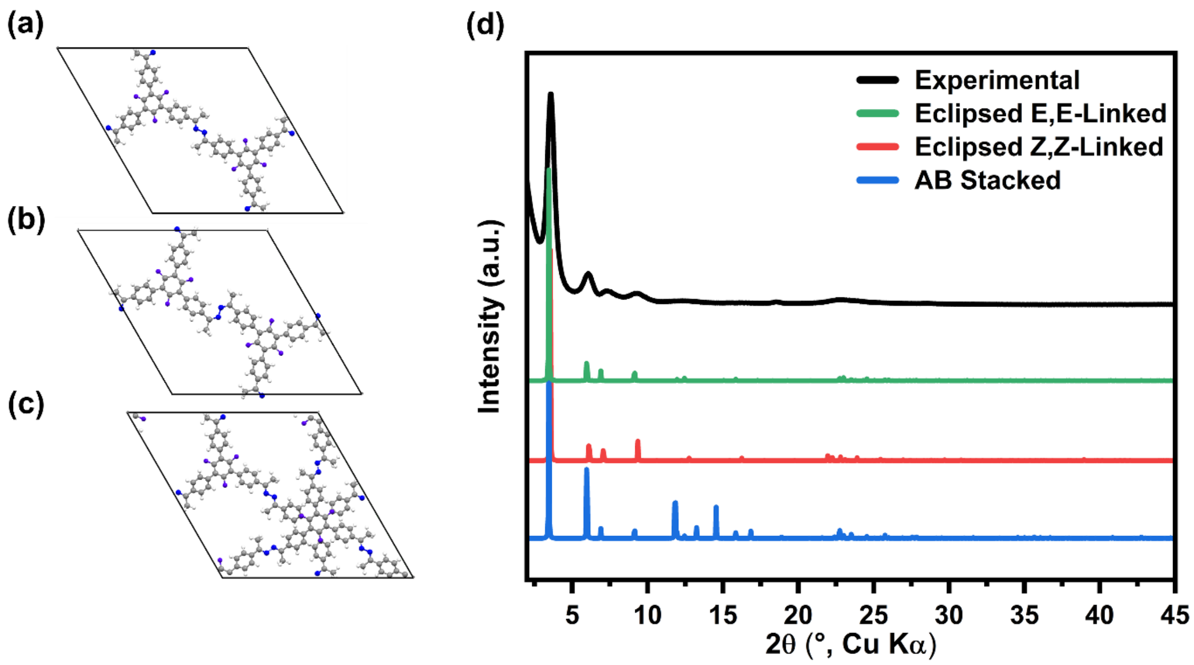

Figure S10. Simulated models of F-Ketazine COF showing (a) eclipsed, (b) eclipsed but Z,Z-conformed ketazine, (c) AB-stacked and (d) respective calculated PXRD patterns compared with experimental pattern

Table S2. Cell parameters and atomic coordinates of F-Ketazine COF

**Space Group**					*P*6, No. 168
**Crystal system**					hexagonal
**Cell parameters**					$a = b = 29.5765$<br>$c = 3.9091$, $\alpha = \beta = 90°$<br>$\gamma = 120°$
**Atom site occupancy**					
**Name**	**Element**	**x**	**y**	**z**	**Occupancy**
C1	C	0.37021	0.85285	0.38020	1
C2	C	0.34964	0.79971	0.38877	1
C3	C	0.37459	0.77626	0.57279	1
C4	C	0.42212	0.81195	0.74353	1
C5	C	0.44221	0.86497	0.72662	1
C6	C	0.41713	0.88766	0.54744	1
C7	C	0.43949	0.94415	0.53456	1
N8	N	0.48940	0.97289	0.58717	1
C9	C	0.40383	0.96696	0.45938	1

H10	H	0.34911	0.86735	0.22526	1
H11	H	0.31387	0.77533	0.24095	1
H12	H	0.44319	0.79732	0.89810	1
H13	H	0.47872	0.88958	0.86696	1
H14	H	0.36256	0.93697	0.42783	1
H15	H	0.41604	0.99061	0.22442	1
H16	H	0.40513	0.99249	0.66877	1
C17	C	0.35386	0.72117	0.58082	1
C18	C	0.29950	0.68712	0.58225	1
F19	F	0.26701	0.70682	0.58081	1

Table S3. Cell parameters and atomic coordinates of n-Ketazine COF

**Space Group**					*P*6, No. 168
**Crystal system**					hexagonal
**Cell parameters**					$a = b = 29.6127$<br>$c = 3.8511$, $\alpha = \beta = 90°$<br>$\gamma = 120°$
**Atom site occupancy**					
**Name**	**Element**	**x**	**y**	**z**	**Occupancy**
C1	C	0.63251	0.14807	0.67502	1
C2	C	0.65322	0.20105	0.64813	1
C3	C	0.62553	0.22286	0.47968	1
C4	C	0.57563	0.18649	0.34112	1
C5	C	0.55581	0.13369	0.37064	1
C6	C	0.58326	0.11221	0.53695	1
C7	C	0.56087	0.05588	0.56613	1
N8	N	0.51059	0.02708	0.53502	1
C9	C	0.59686	0.03320	0.63184	1
H10	H	0.65565	0.13471	0.81931	1
H11	H	0.69111	0.22688	0.77072	1
H12	H	0.55276	0.20068	0.20150	1
H13	H	0.51738	0.10824	0.25313	1

H14	H	0.63839	0.06302	0.64400	1
H15	H	0.59315	0.00596	0.42461	1
H16	H	0.58692	0.01137	0.87739	1
C17	C	0.70030	0.31339	0.45466	1
C18	C	0.72175	0.36794	0.45845	1
H19	H	0.72688	0.29764	0.46133	1

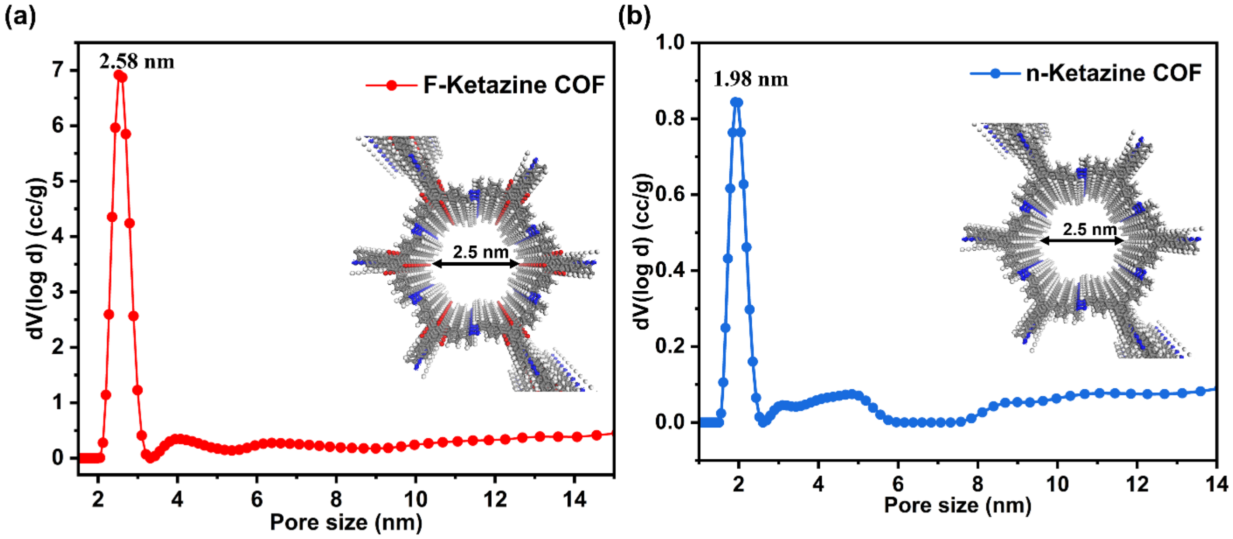

Figure S11. Pore size distribution of (a) F-Ketazine COF, (b) n-Ketazine COF calculated from the adsorption branch of isotherms by the QSDFT model.

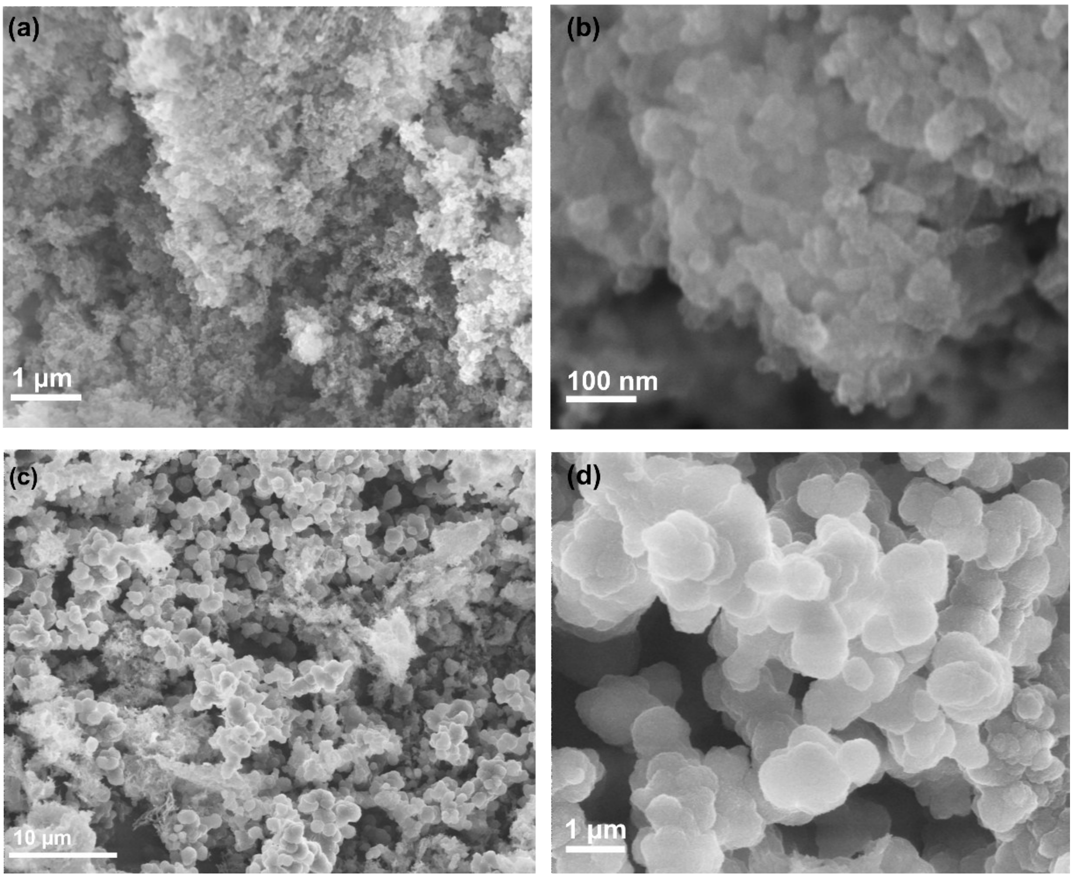

Figure S12. FESEM images of (a,b) F-Ketazine COF, (c,d) n-Ketazine COF.

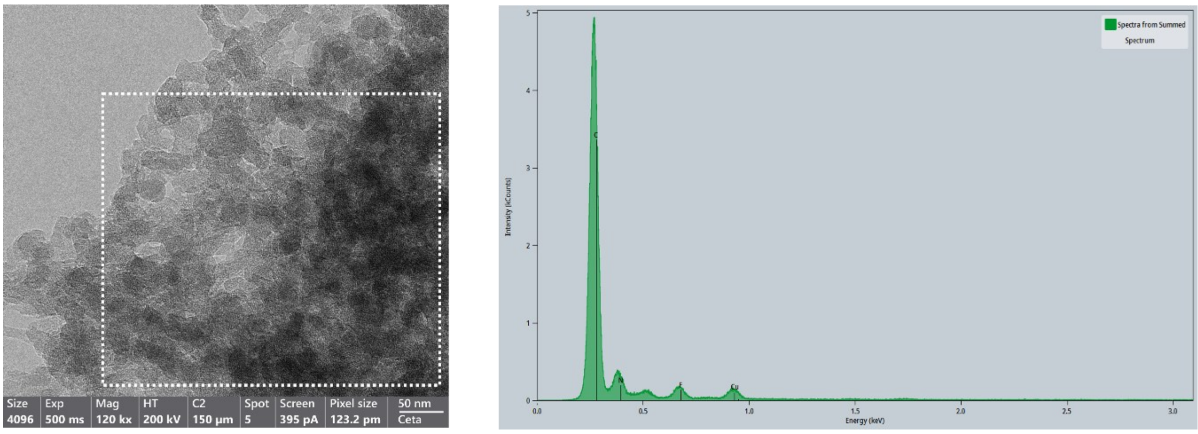

Figure S13. Low-dose HRTEM images of F-Ketazine COF. The specific region analyzed by EDX is highlighted by the white square. No metal was detected within this area, besides a minor Cu signal, which arises from the copper TEM grid.

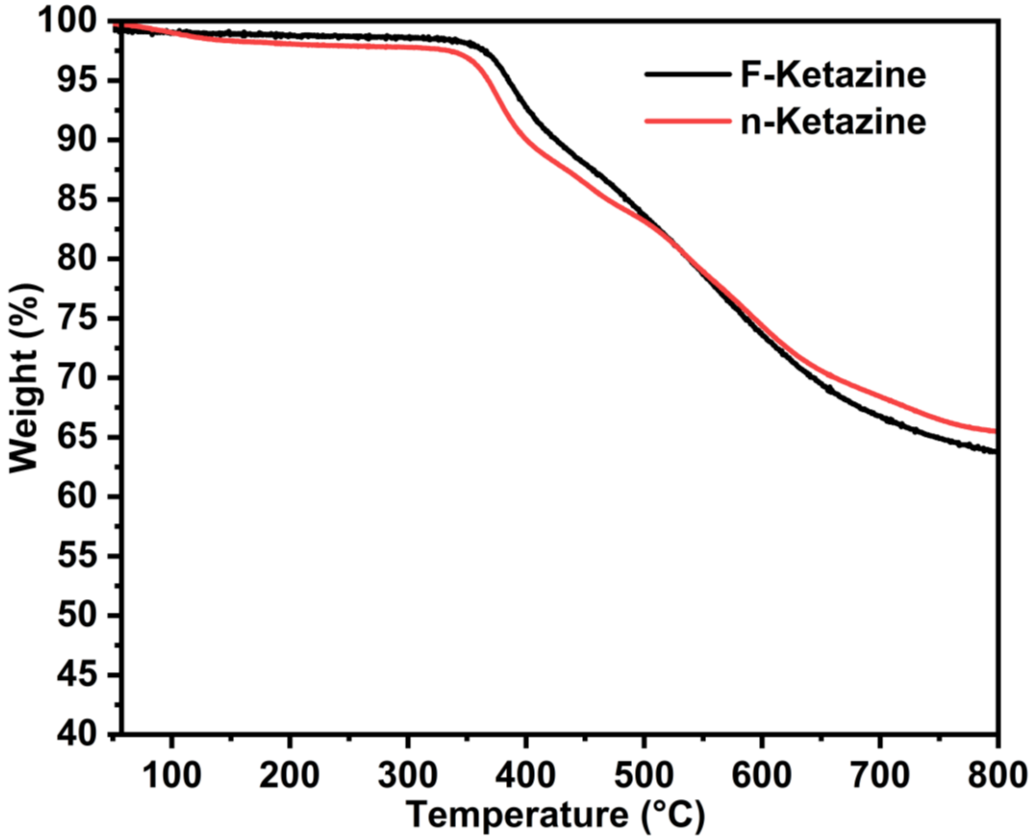

Figure S14. TGA data of as-synthesized F-Ketazine, and n-Ketazine COFs under N_2 atmosphere.

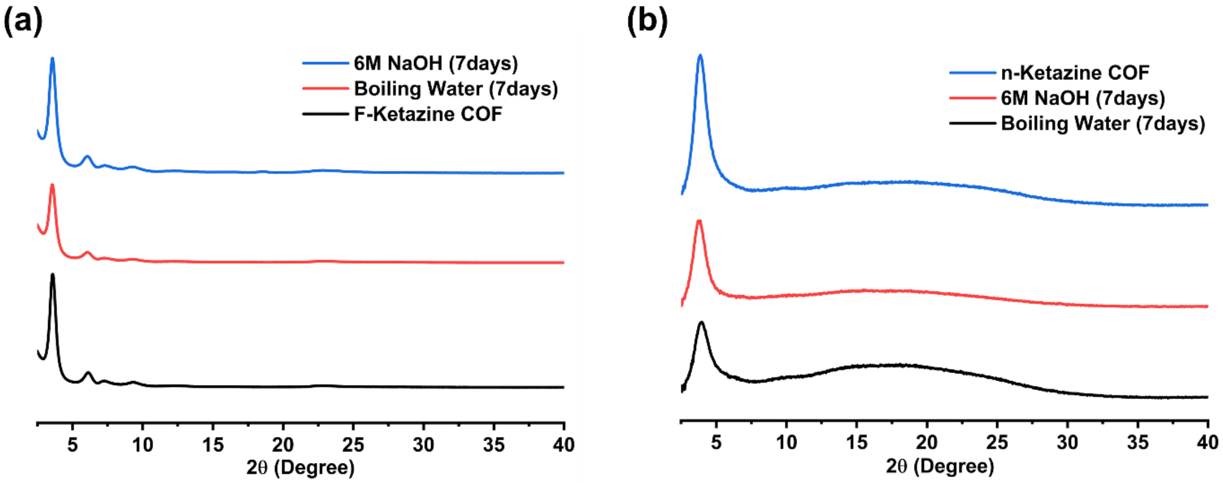

Figure S15. PXRD of (a) F-Ketazine COF, (b) n-Ketazine COF after treatment with boiling water, 6M NaOH for 7days.

Chemical stability was evaluated by immersing the COF powders for both COFs in 6 M HCl, 6 M NaOH, and boiling water for 7 days. After treatment, the samples were thoroughly rinsed with deionized water, washed with acetone, dried, and analyzed by PXRD. Both COFs retained their crystallinity after exposure to 6 M NaOH and boiling water, indicating excellent structural

stability under alkaline and hydrothermal conditions. In contrast, samples exposed to 6 M HCl fully dissolved during the acetone washing step, leaving no solid material for PXRD analysis.

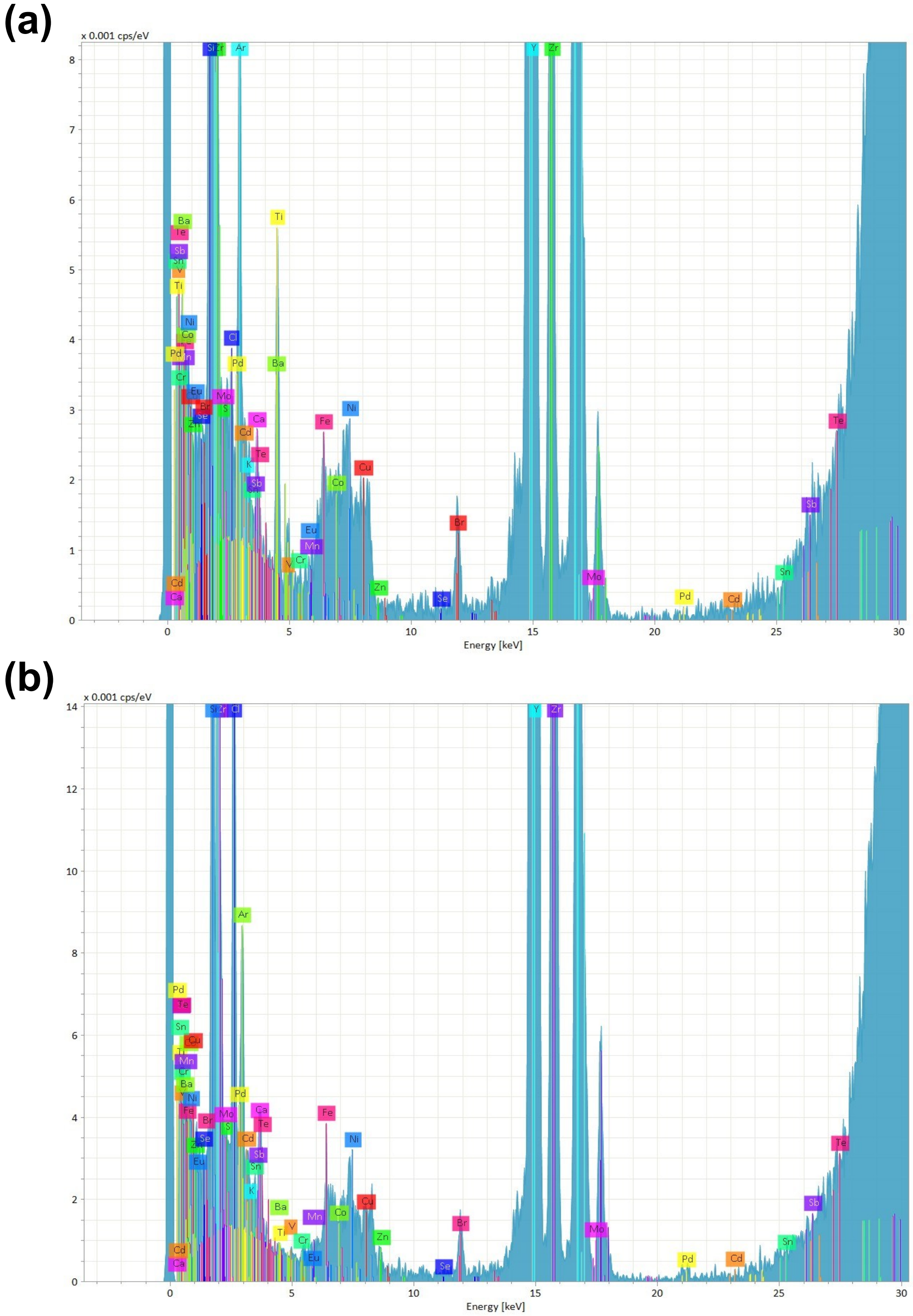

Figure S16. Representative TXRF spectra of (a) aqua regia, (b) F-Ketazine COF+aqua regia, employed for for quantitative determination of the Pd content.

Table S4. Pd content as derived from TXRF

Materials	Pd mg/l	Pd Wt%
F-Ketazine COF	0.066 ± 0.007	0.00384± 0.0008 %
Aqua regia	0.018 ± 0.007	$1.63 \pm 0.6 \times 10^{-6}$ %

Metal Content based quantification of F-Ketazine COF by using ICP-OES,

Dilution Process:

Initial catalyst mass: 10 mg dissolved in 4 mL of aqua regia.

Aliquot taken: 330 µL from the initial solution.

Dilution: The aliquot was diluted with 2.5 mL of water, resulting in a final volume of 2830 µL (2.83 mL).

Dilution factor (DF) = final volume / aliquot volume = 2830 µL / 330 µL = 8.5758.

Calculation Formula:

The weight percentage (wt%) of Pd in the catalyst is calculated using:

$$\text{wt\% Pd} = C_{ICP} \times DF \times \frac{Vorg}{10} \times 100$$

Where: C_{ICP} is the ICP concentration of Pd in mg/L,

DF is the dilution factor (8.5758),

V_{org} is the volume of the original solution (4 mL = 0.004 L),

The catalyst mass is 10 mg.

Simplifying:

$$\text{wt\% Pd} = C_{ICP} \times 8.5758 \times 0.004 \times 10$$

$$= C_{ICP} \times 0.34303$$

Results:

ICP concentration (C_{ICP}): 0.0090641 mg/L

wt% Pd = 0.0090641 × 0.34303 =0.003109 %

Alternatively, in parts per million (ppm): 31.09 ppm (since 1% = 10,000 ppm).

Estimation of Maximum Possible Current Contribution from Residual Pd

The F-Ketazine COF contains residual Pd at 38 ppm (0.0038 wt%) as determined by ICP-OES/TXRF. The catalyst loading on the working electrode is 0.15 mg cm^{-2}. Therefore, the absolute mass of Pd on the electrode is:

Mass of Pd= 0.15 mg cm^{-2} × 38 ×10^{-6} = 5.7×10^{-6} mg cm^{-2} = 5.7 ng cm^{-2}

This Pd loading is several orders of magnitude lower than typical Pd-based catalysts reported for NO_3RR, which generally employ loadings in the µg to mg cm^{-2} range. Even if 100% of this trace Pd were electrochemically active, the theoretical maximum current contribution would be negligible (nA to low µA range) compared to the measured current densities (mA range) achieved by the metal-free F-Ketazine COF.

This calculation, combined with post-electrolysis XPS (showing no Pd 3d signal) and TEM (showing no Pd nanoparticles), confirms that residual Pd does not contribute to the observed catalytic activity.

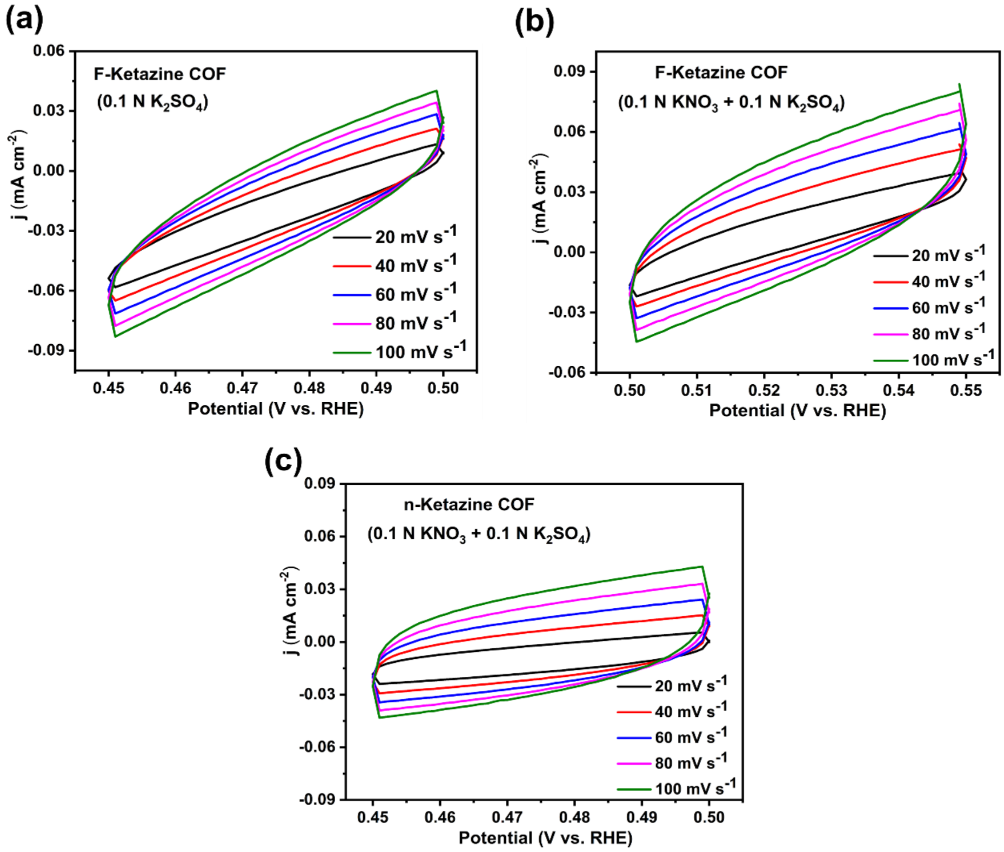

Figure S17. CV diagrams of the catalyst at non-faradaic region (a) F-Ketazine COF in absence of nitrate solution, (b) F-Ketazine COF in presence of nitrate solution, (c) n-Ketazine COF in presence of nitrate solution.

Table S5: Raw data for double-layer capacitance

Catalyst	Scan rate (mV/s)	Δj/2 (mA/cm^2)	
		**Test-1**	**Test-2**
**F-Ketazine COF**	20	0.0096	0.0094
	40	0.01615	0.0161
	60	0.0218	0.0211
	80	0.0272	0.0271
	100	0.0328	0.0331
**n-Ketazine COF**	20	0.0093	0.0088
	40	0.0138	0.0131
	60	0.01905	0.0192
	80	0.0248	0.02435
	100	0.03	0.02955

Catalyst	C_{dl} (mF/cm^2) Test-1	C_{dl} (mF/cm^2) Test-2	Mean ± SD (n=2)
F-Ketazine COF	0.287	0.292	0.2895 ± 0.0035
n-Ketazine COF	0.262	0.264	0.2630 ± 0.0014

Raw data for double-layer capacitance (C_{dl}) determination. Two independent replicate tests are shown for each catalyst in nitrate-containing electrolyte. Δj/2 (half-difference of capacitive current density) was measured at scan rates from 20 to 100 mV/s. The C_{dl} values were obtained from the slope of Δj/2 vs. scan rate for each test. The mean ± standard deviation (n=2) is provided for clarity.

Table S6 - Linear regression statistics for C_{dl} determination

Catalyst	Slope	Slope	**Statistics**
	Value (F cm^{-2})	Standard Error	Adj. R-Square
F-Ketazine COF (without nitrate)	2.12×10^{-4}	4.34×10^{-6}	0.99832
F-Ketazine COF (containing nitrate)	2.87×10^{-4}	6.14×10^{-6}	0.99818
n-Ketazine COF (containing nitrate)	2.62×10^{-4}	6..03×10^{-6}	0.99789

Table S7. Various data sheet on resistance at various potential using two different COF systems

Sl. No.	Catalyst	Potential (V vs. RHE)	R_s (Solution Resistance)	R_{ct} (Charge Transfer Resistance)
1	n-Ketazine COF	0V	~1	3589
2		–0.5V	~1	3.676
3		–0.7V	~1	2.041
4		–0.9V	~1	1.475
1	F-Ketazine COF	0V	~1	706.4
2		–0.5V	~1	2.373
3		–0.7V	~1	1.746
4		–0.9V	~1	1.225

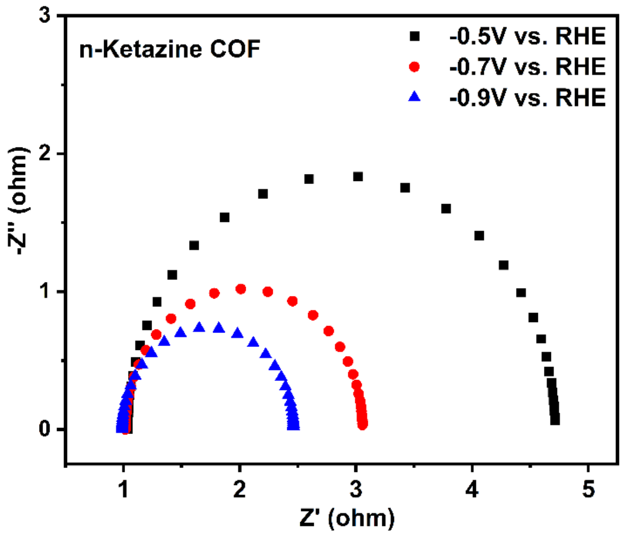

Figure S18. EIS diagram for n-Ketazine COF at various potential

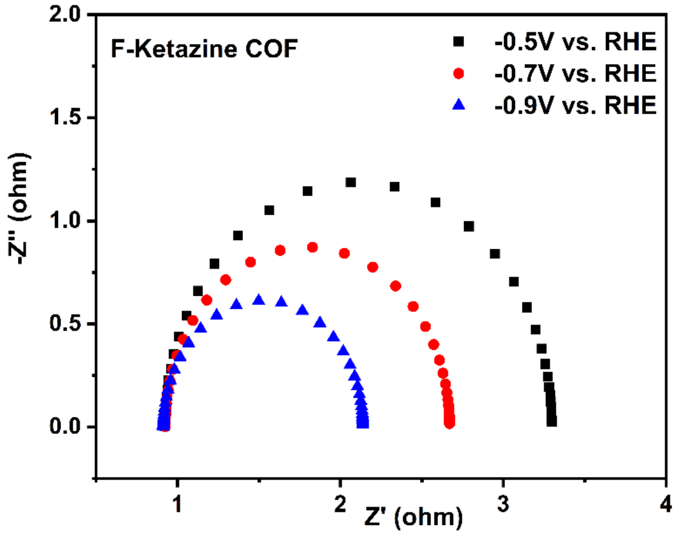

Figure S19. EIS diagram for F-Ketazine COF at various potential

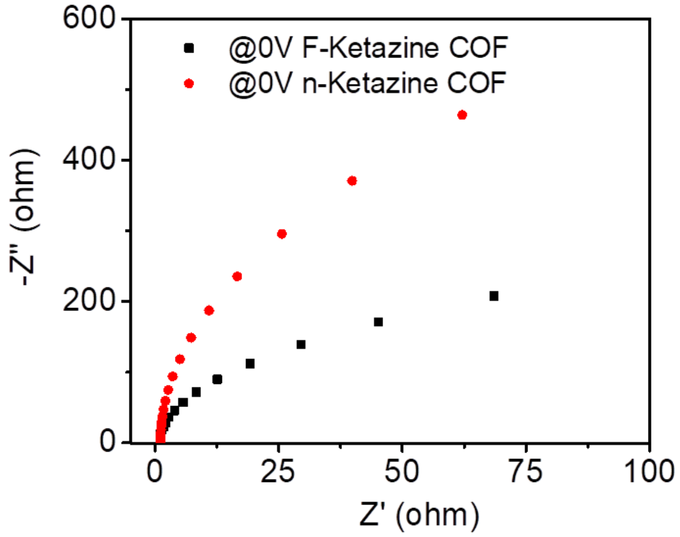

Figure S20. EIS diagram for n-Ketazine and F-Ketazine COF at 0V

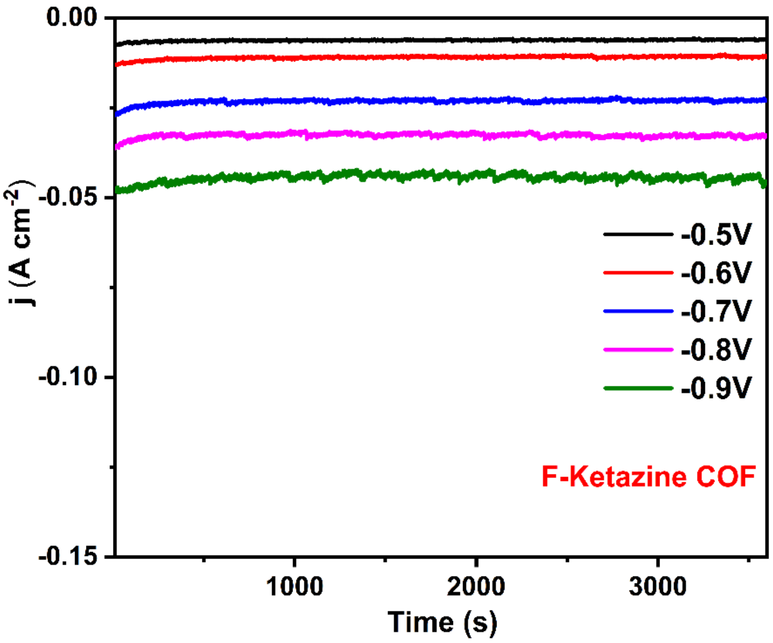

Figure S21. j vs t diagram for 1h of electrolysis using F-Ketazine COF at various potential range

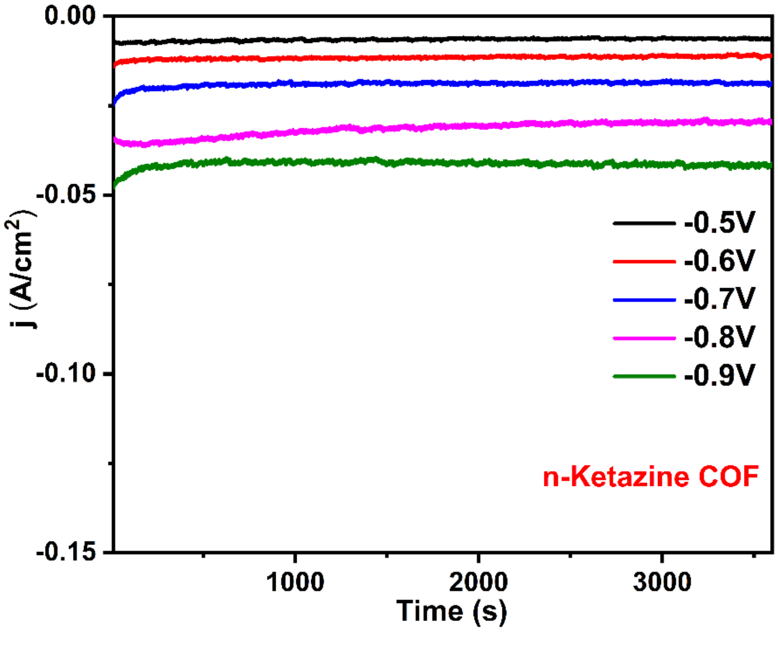

Figure S22. j vs t diagram for 1h of electrolysis using n-Ketazine COF at various potential range

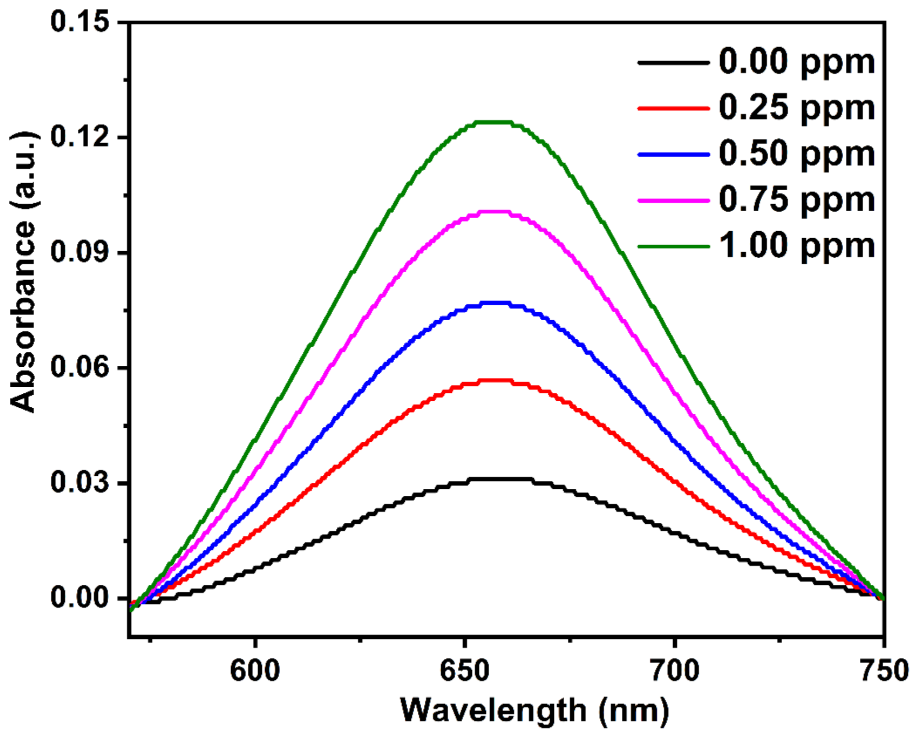

Figure S23. Standard ammonia calibration curve at various concentration ranges

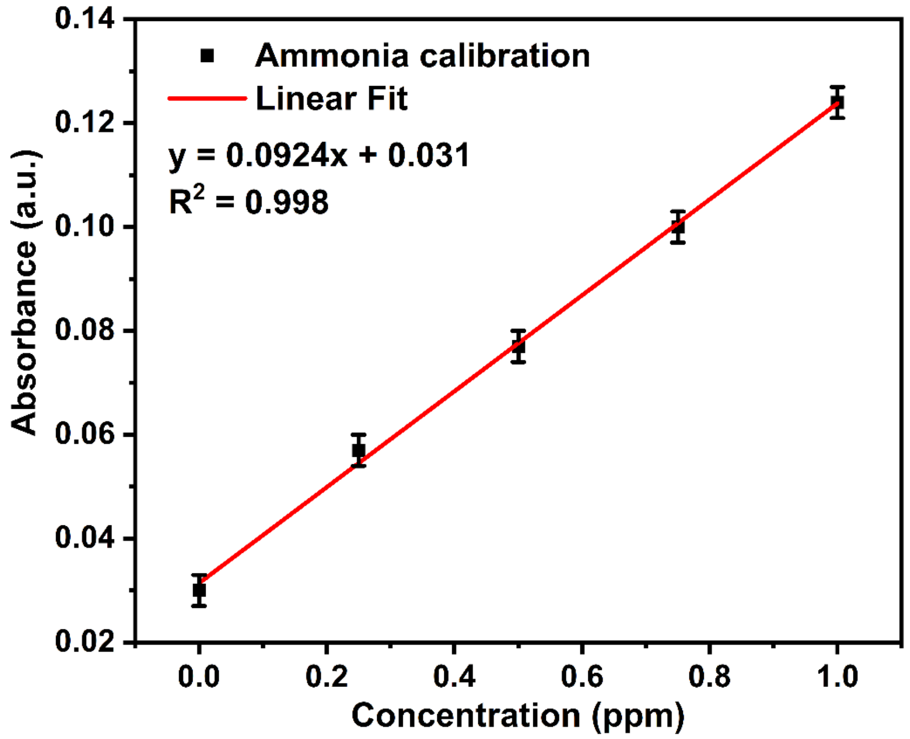

Figure S24. Standard ammonia straight line calibration curve at various concentration ranges

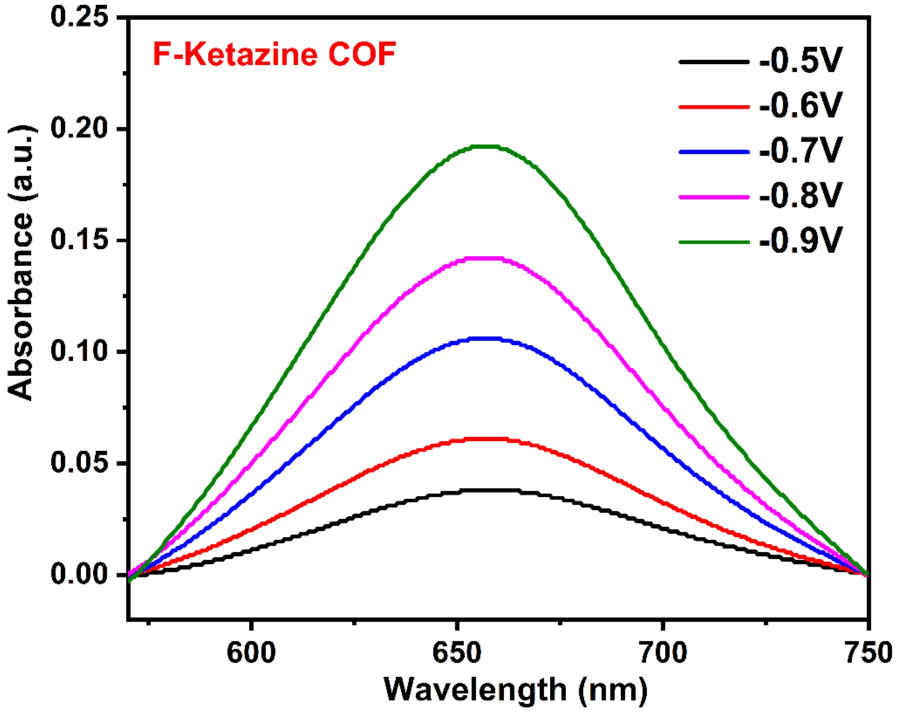

Figure S25. UV plot of electrolyte solution after electrolysis using F-Ketazine COF at various potential ranges

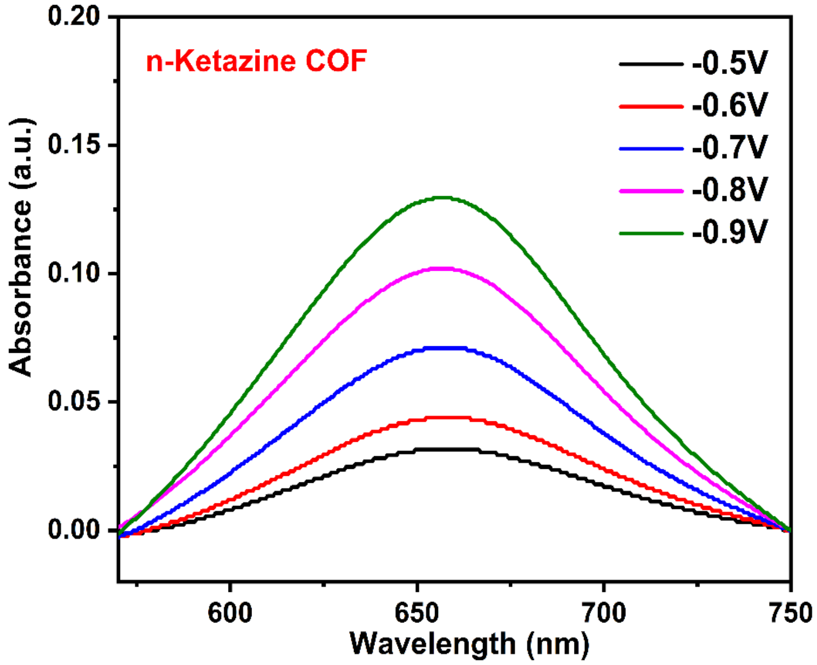

Figure S26. UV plot of electrolyte solution after electrolysis using n-Ketazine COF at various potential ranges

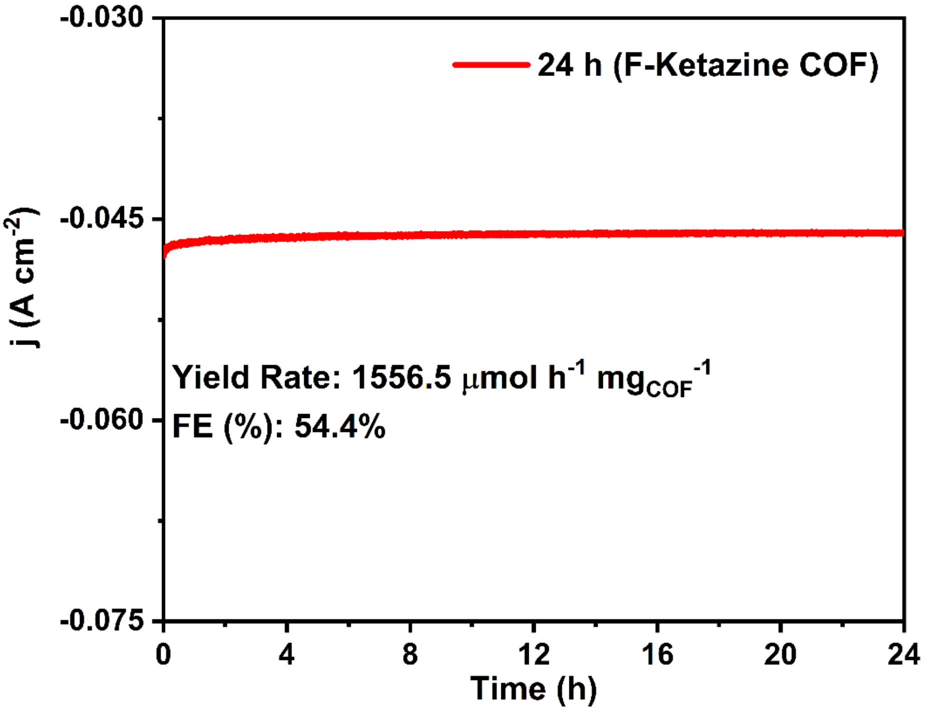

Figure S27. Long term (24h) experiment using F-Ketazine COF

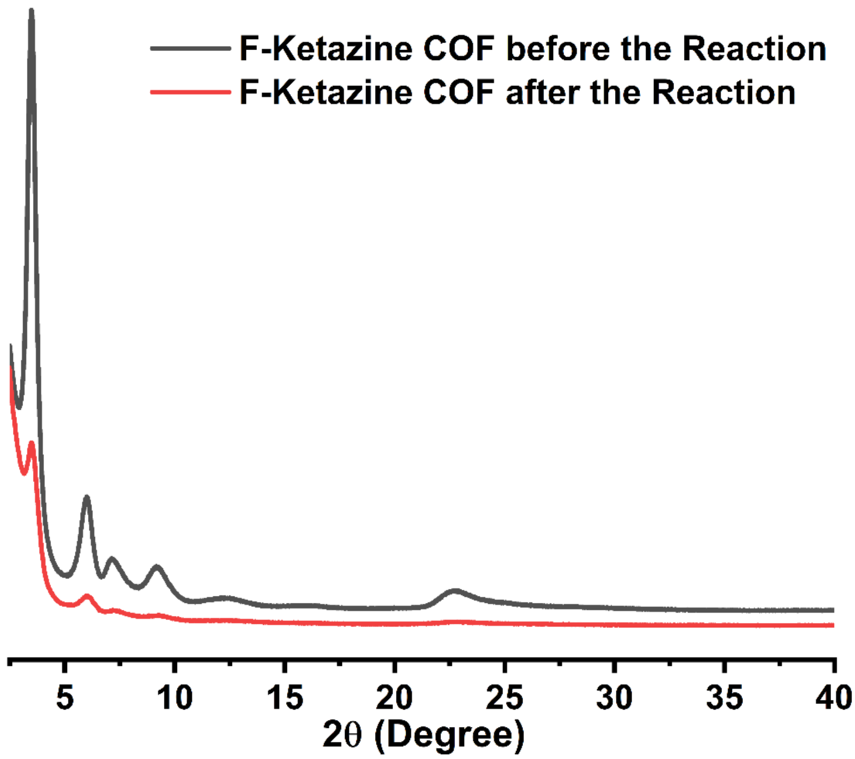

Figure S28. PXRD of F-Ketazine COF before and after the long-term experiment

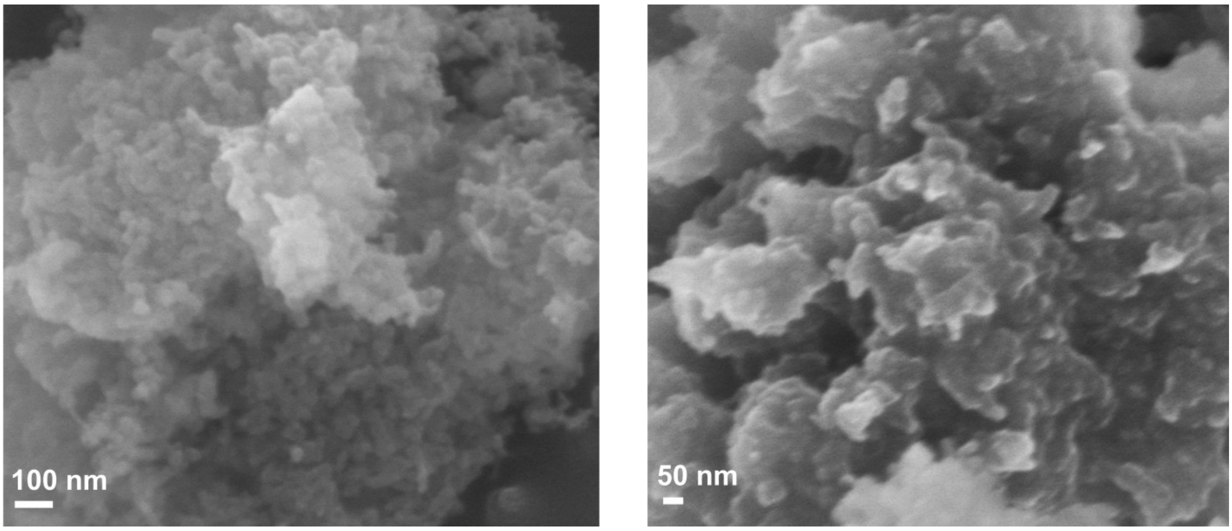

Figure S29. FESEM images of the F-Ketazine COF after the long-term experiment

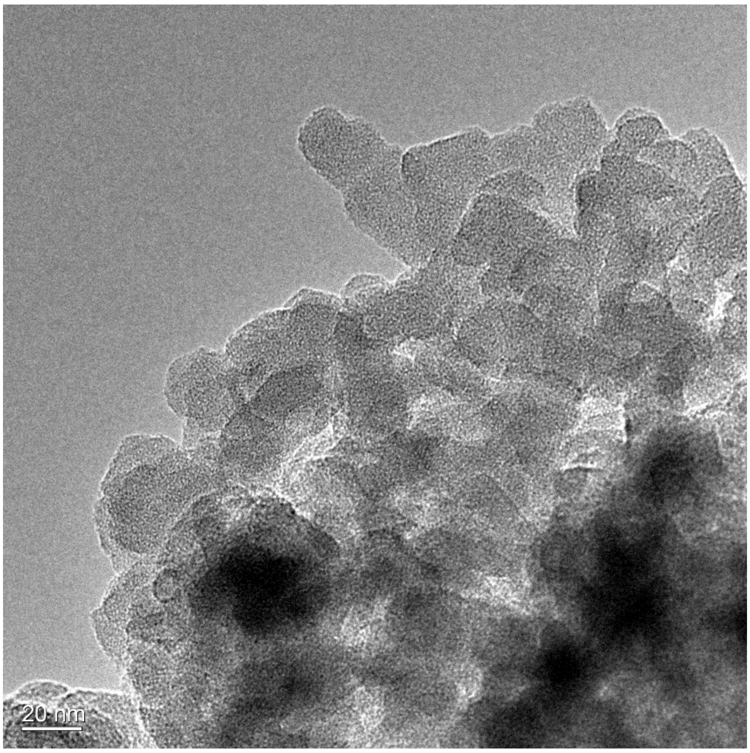

Figure S30. TEM images of the F-Ketazine COF after the long-term experiment.

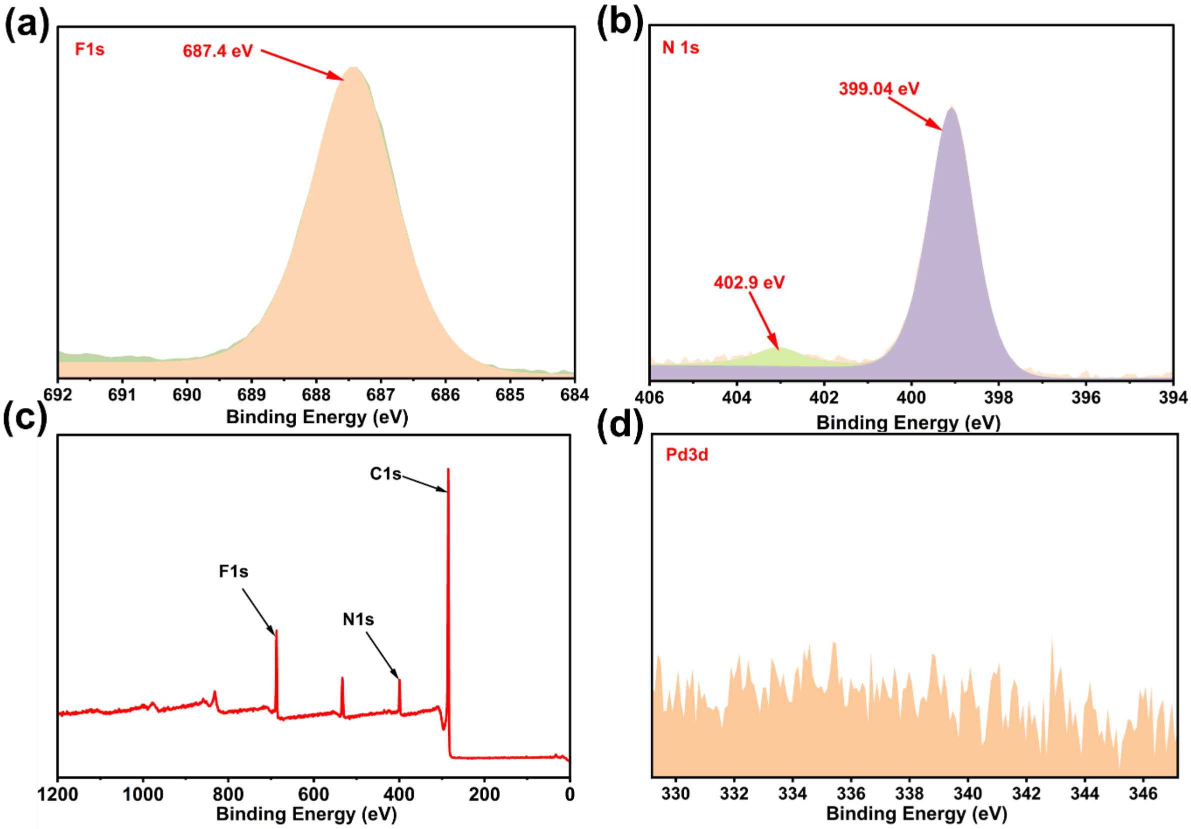

Figure S31. F 1s, N 1s, Pd 3d and XPS survey spectra of F-Ketazine COF after the long-term experiment.

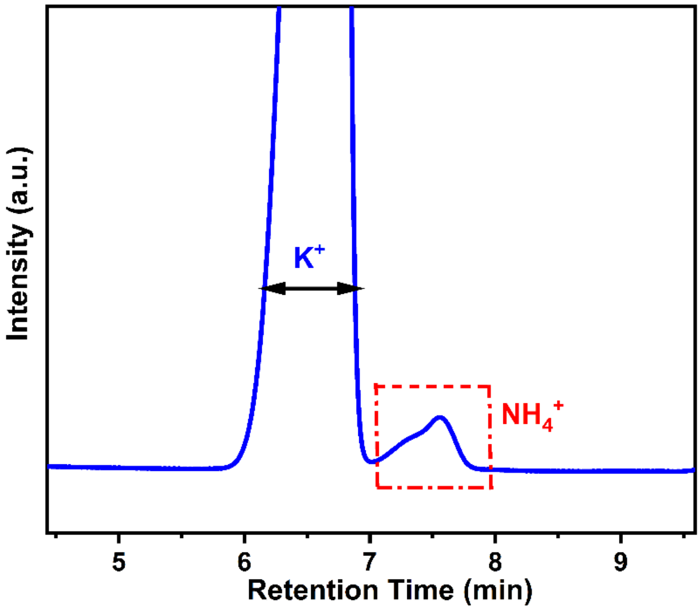

Figure S32. Ion chromatography (IC) analysis (Eco IC Metrohm model, Switzerland) of the electrolyte for detection of ammonium ions (@-0.9V vs. RHE)

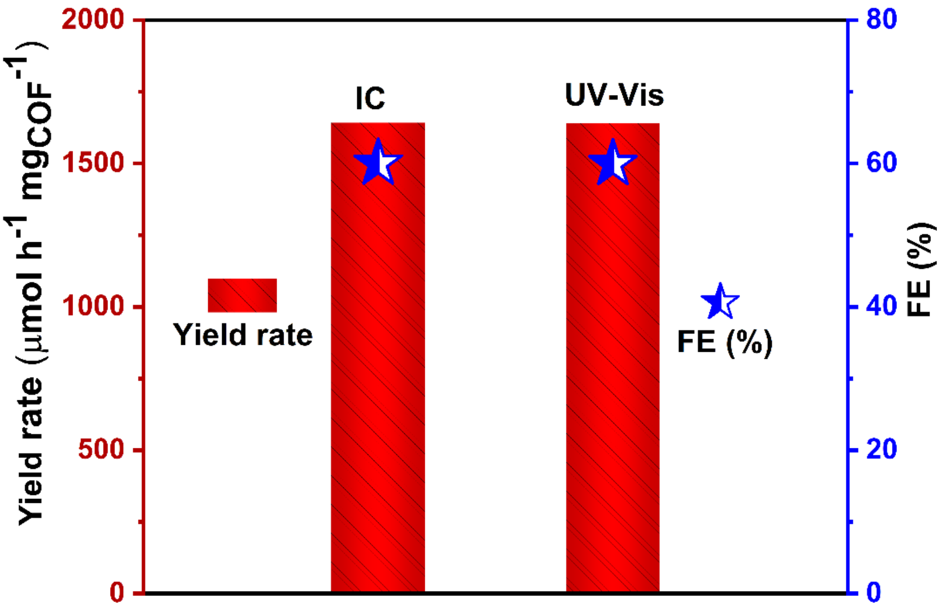

Figure S33. Bar diagram of ammonia yield rate and FE using IC vs. UV-Vis method @-0.9V vs. RHE.

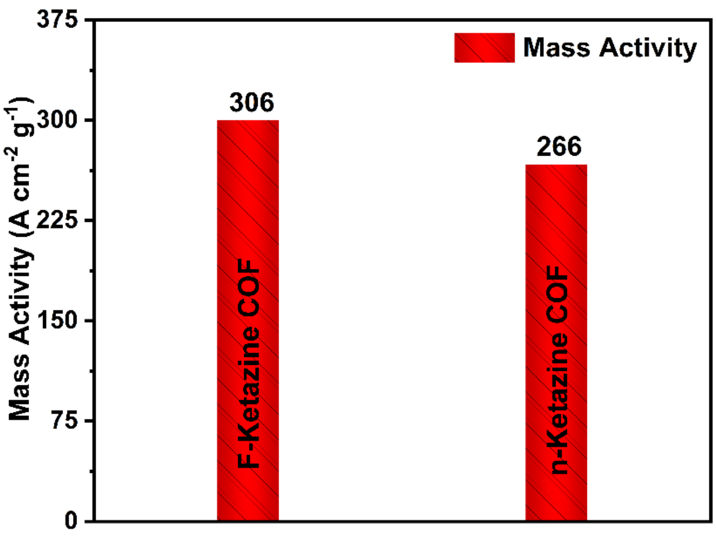

Figure S34. Bar diagram of mass activity @-0.9V vs. RHE using F-Ketazine COF and n-Ketazine COF system

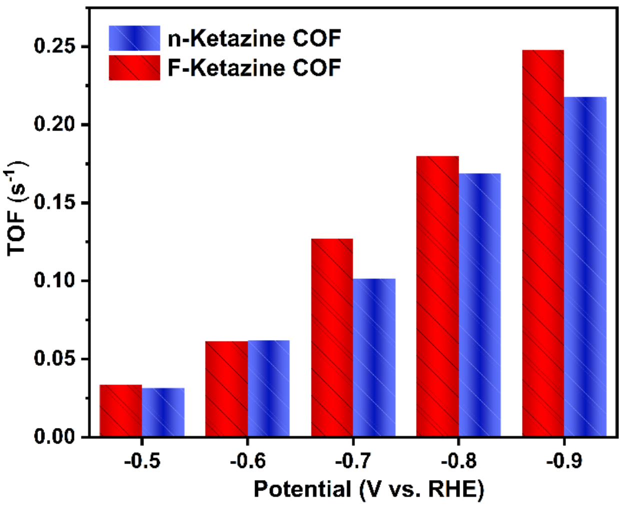

Figure S35. Bar diagram of TOF (s^{-1}) @-0.9V vs. RHE using F-Ketazine COF and n-Ketazine COF system

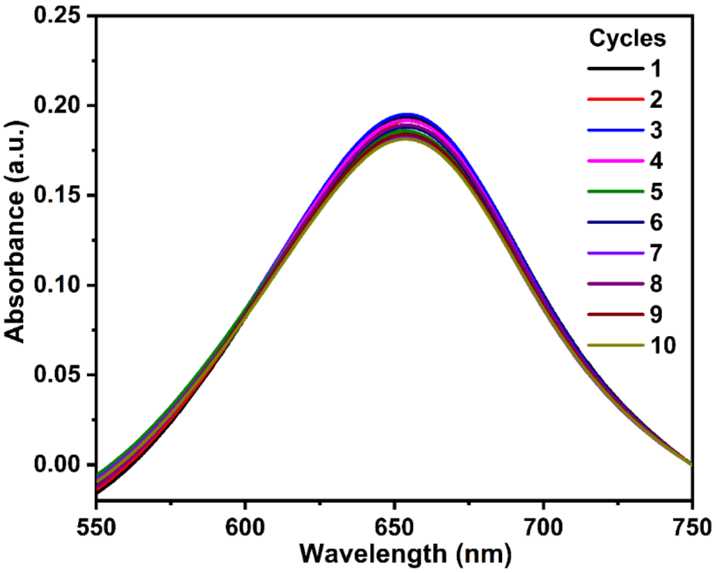

Figure S36. UV plot of electrolyte solution after electrolysis using F-Ketazine COF after 10 cycles

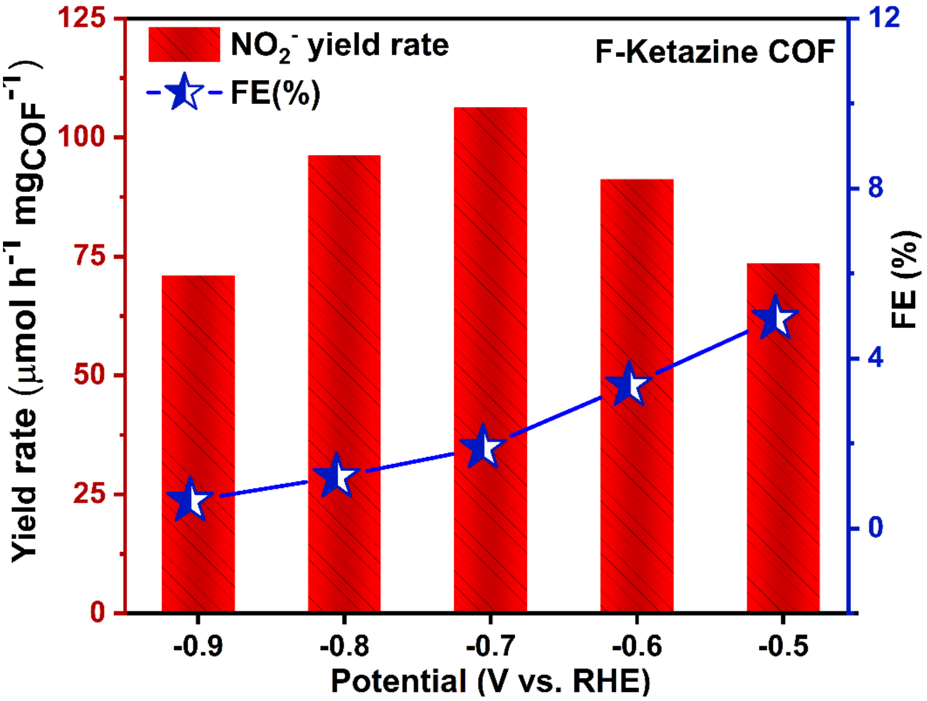

Figure S37. Bar diagram of NO_2^- yield rate and FE at various potential using F-Ketazine COF

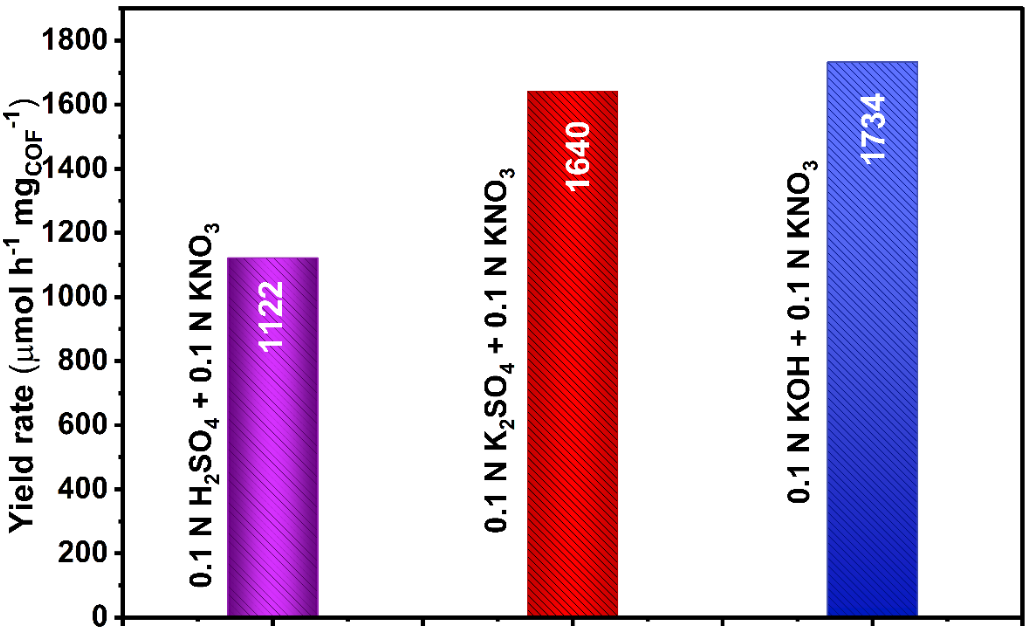

Figure S38. pH control NO_3RR activity in the electrolyte for F-Ketazine COF

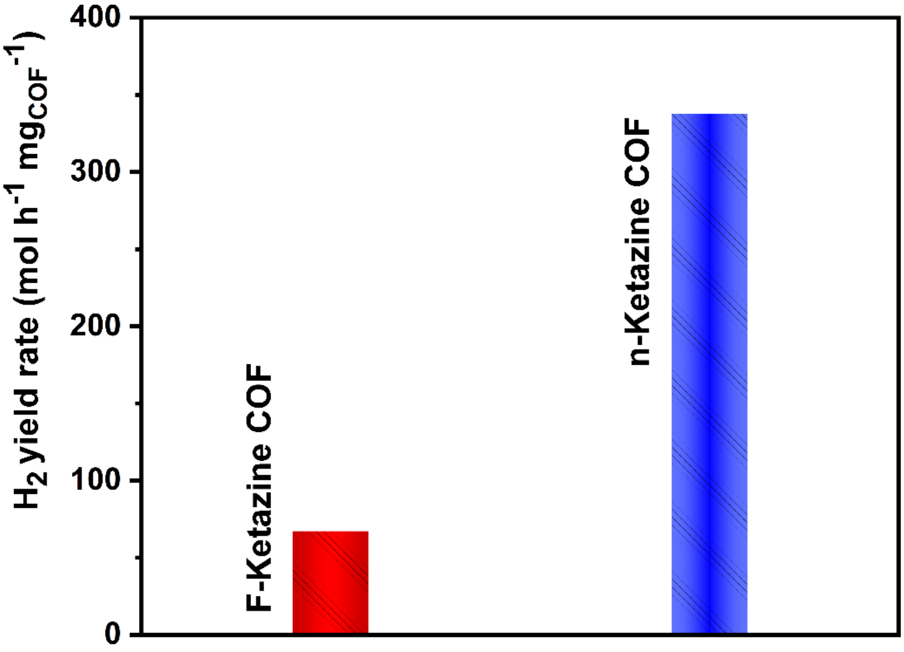

Figure S39. Bar diagram of H_2 production using GC analysis @-0.9V vs. RHE using F-Ketazine COF and n-Ketazine COF system.

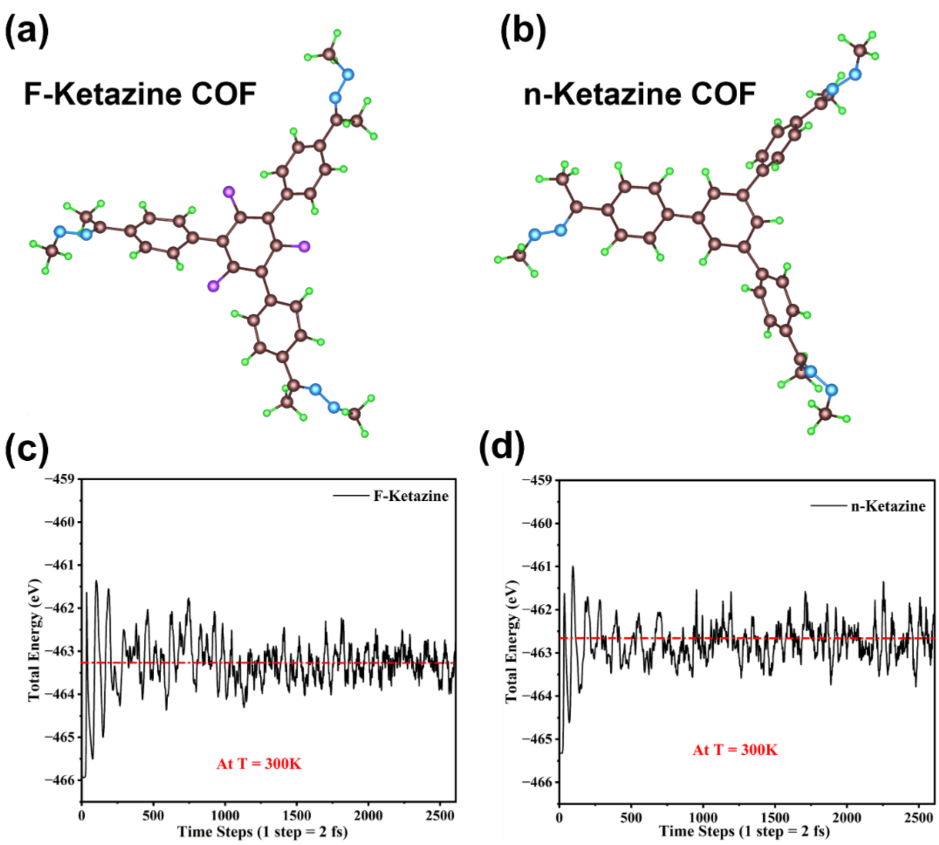

Figure S40. (a) and (b) optimized structures after the AIMD simulations with 5200 fs of time at T = 300 K for F-Ketazine and n-Ketazine catalysts. (c) and (d) plot of total energy of the two Ketazines against time.

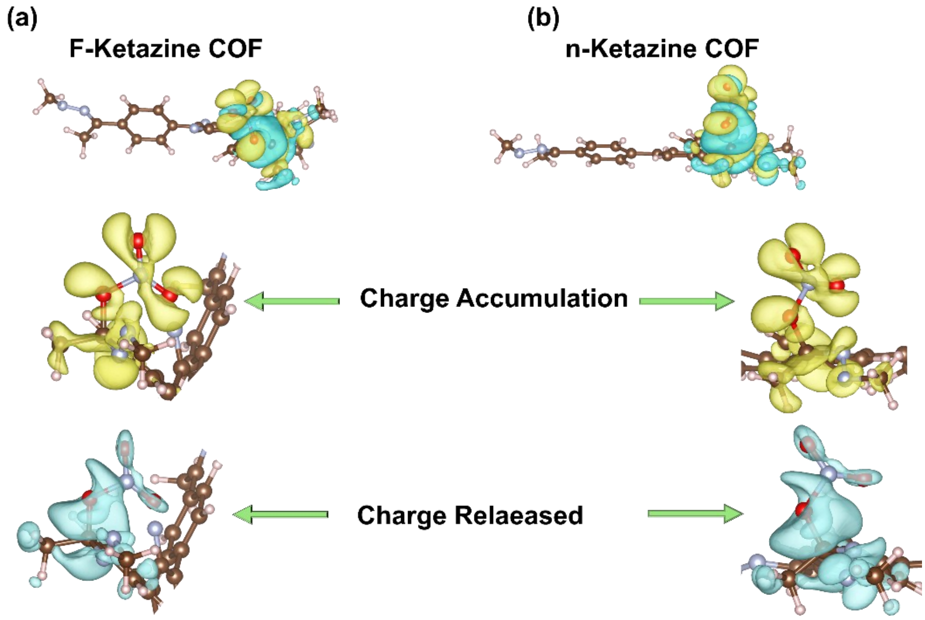

Figure S41. (a) and (b) charge density difference plots of adsorbed NO_3^- molecule for F-Ketazine and n-Ketazine COF.

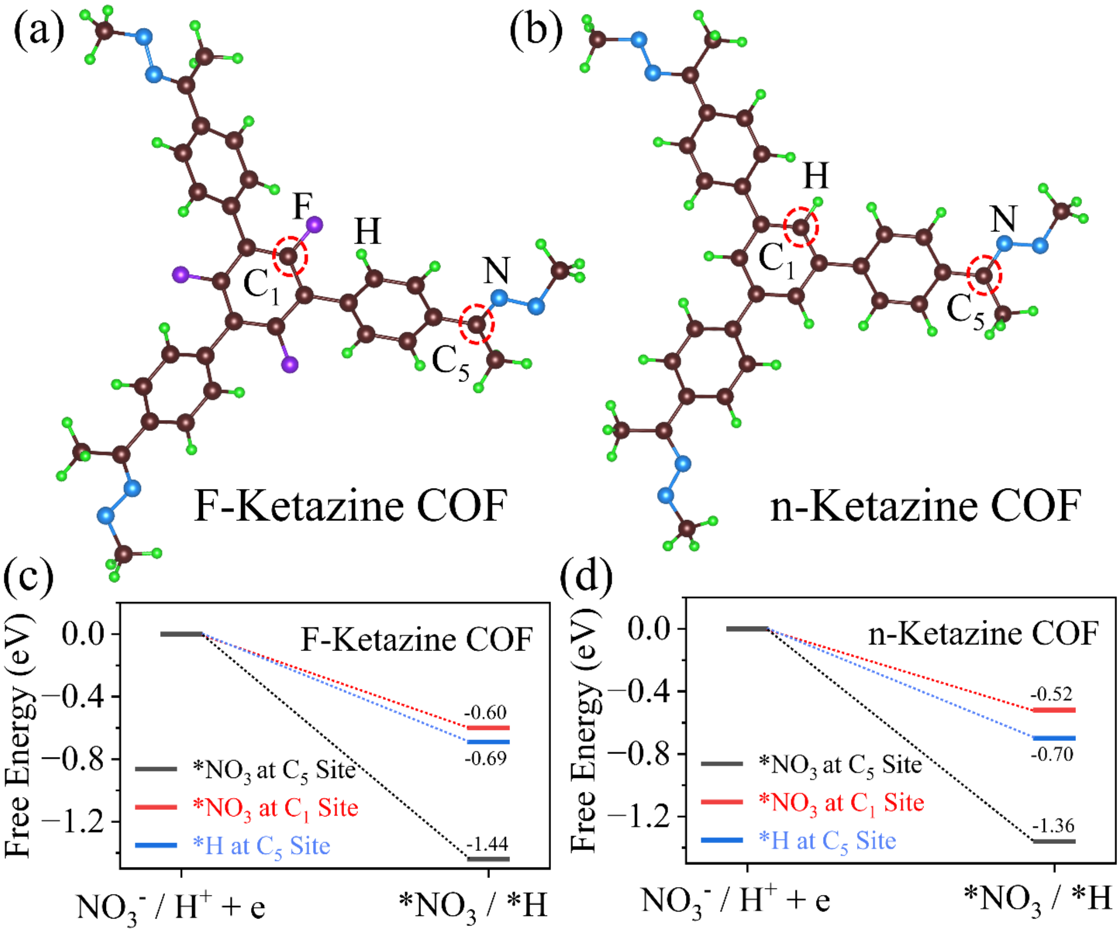

Figure S42. (a) and (b) optimized structures of F-Ketazine and n-Ketazine COF, (c) and (d) comparison of NO_3- and H-binding for F-Ketazine and n-Ketazine COF.

Table S8. The comparison of electrocatalytic NO_3RR performance of metal-free F-Ketazine linked COF with other reported catalysts.

Catalyst	Electrolyte	NH_3 Yield rate	FE (%)	Ref.
F-Ketazine COF	0.1 N KNO_3/ 0.1 N K_2SO_4	1639.9 μmol h^{-1} mg_{COF}^{-1} at -0.9 V vs. RHE	59.9% at -0.9 V vs. RHE	This Work
n-Ketazine COF	0.1 N KNO_3/ 0.1 N K_2SO_4	1113.4 μmol h^{-1} mg_{COF}^{-1} at -0.9 V vs. RHE	41% at -0.9 V vs. RHE	This Work
Py-PyIM-Fe	1M KOH/0.1 M KNO_3	4299.61 μmol h^{-1} mg_{COF}^{-1} at -2 V vs. SCE	86.9% at -2 V vs. SCE	[12]
COF-366-Fe	0.5 M K_2SO_4/0.1 M KNO_3	1883.6 μmol h^{-1} mg_{COF}^{-1} at -1.7V vs. SCE	85.4% at -1.7 V vs. SCE	[13]
Cu SAGs	0.1 M PBS/20 mM NO_3	0.44 mg h^{-1} cm^{-2} at -0.8 V vs. RHE	78% at -0.8 V vs. RHE	[14]
NiPr-TPA-COF	0.5 M K_2SO_4/0.1 M KNO_3	2.5 mg h^{-1} cm^{-2} at -1.46V vs. SCE	90% at -1.46 V (vs. SCE)	[15]
Cu/JDC/CP	0.1 M NaOH/0.1 M NO_2	0.52 mmol h^{-1} mg^{-1}_{cat} at -0.6 V vs. RHE	93.2% at -0.6 V vs. RHE	[16]
Fe−N−C	0.05 M PBS/0.16 M NO_3	10 μmol h^{-1} cm^{-2} at -0.45 V vs. RHE	82% at -0.45 V vs. RHE	[17]
FeMo-N-C	0.05 M PBS/0.16 M NO_3	17 μmol h^{-1} cm^{-2} at -0.45 V vs. RHE	93% at -0.45 V vs. RHE	[18]
Fe SAC	0.1 M K_2SO_4/0.5 M KNO_3	5.24 mg h^{-1} mg^{-1}_{cat} at -0.66 V vs. RHE	75% at -0.66 V vs. RHE	[19]

To date, all reported COF-based NO_3RR catalysts utilize metal centers. This work presents the first metal-free COF for NO_3RR, establishing a baseline for future development of sustainable organic electrocatalysts.

Table S9. Comparison of structural and electrochemical metrics for F-Ketazine and n-Ketazine COFs.

Catalyst	BET Surface Area (m^2 g^{-1})	Yield (μmol h^{-1} mg_{COF}^{-1})	Faradaic efficiency (%)	TOF (S^{-1})	C_{dl} (mF cm^{-2})	ECSA (cm^2)	Mass activity (A g^{-1})
F-Ketazine COF	1550	1639.9 at -0.9 V vs. RHE	59.9	0.25	0.287	14.35	306
n-Ketazine COF	220	1113.4 at -0.9 V vs. RHE	41	0.21	0.262	13.10	266

ECSA calculation:[20,21]

From the C_{dl} values, we determined the electrochemical active surface area (ECSA).

ECSA = R_f x S

Where R_f is the roughness factor and S is the geometrical surface area of the electrode (1 x 1 cm^2 catalyst loaded on the both side of carbon paper).

$R_f = (C_{dl}$ mF cm^{-2}/ 40 μF $cm^{-2})$

Specific capacitance of the flat surface in generally 40 μF cm^{-2}

Table S10. NO_3^- binding energies and limiting potentials for Ketazine systems with varying fluorine content.

System	NO_3 binding energy (eV)	Limiting Potential (V)
F-Ketazine with 3 F-atoms	-1.44	0.61
F-Ketazine with 2 F-atoms	-1.48	0.61
F-Ketazine with 1 F-atoms	-1.44	0.60
n-Ketazine with no F-atoms	-1.36	0.65

References

(1) Limbeck, A.; Bonta, M.; Nischkauer, W. Improvements in the Direct Analysis of Advanced Materials Using ICP-Based Measurement Techniques. *Journal of Analytical Atomic Spectrometry* 2017, *32*, 212–232.

(2) Ruickoldt, J.; Kreibich, J.; Bick, T.; Jeoung, J. H.; Duffus, B. R.; Leimkühler, S.; Dobbek, H.; Wendler, P. Ligand Binding to a Ni–Fe Cluster Orchestrates Conformational Changes of the CO-Dehydrogenase–Acetyl-CoA Synthase Complex. *Nature Catalysis* 2025, *8*, 657–667.

(3) Kresse, G. *Ab Initio Molecular-Dynamics Simulation of the Liquid-Metal-Amorphous-Semiconductor Transition in Germanium*; Vol. 8.

(4) Kresse, G.; Hafner, J. *Ab. Initio Molecular Dynamics for Liquid Metals*; Vol. 47.

(5) Kresse, G.; Hafner, J. *Ab Initio Molecular Dynamics for Open-Shell Transition Metals*; 1993; Vol. 48.

(6) Kresse, G.; Joubert, D. *From Ultrasoft Pseudopotentials to the Projector Augmented-Wave Method*.

(7) Perdew, J. P.; Burke, K.; Ernzerhof, M. *Generalized Gradient Approximation Made Simple*; 1996.

(8) Grimme, S.; Antony, J.; Ehrlich, S.; Krieg, H. A Consistent and Accurate Ab Initio Parametrization of Density Functional Dispersion Correction (DFT-D) for the 94 Elements H-Pu. *Journal of Chemical Physics* 2010, *132* (15). https://doi.org/10.1063/1.3382344.

(9) Han, X.; Zhou, Z.; Wang, K.; Zheng, Z.; Neumann, S. E.; Zhang, H.; Ma, T.; Yaghi, O. M. Crystalline Polyphenylene Covalent Organic Frameworks. *Journal of the American Chemical Society* 2024, *146*, 89–94.

(10) *Fluorine | XPS Periodic Table | Thermo Fisher Scientific - EN*. https://www.thermofisher.com/de/de/home/materials-science/learning-center/periodic-table/halogen/fluorine.html?utm_source=chatgpt.com (accessed 2025-09-28).

(11) *Fluorine (F), Z=9, & Fluorine Compounds*. https://xpsdatabase.net/fluorine-f-z9-fluorine-compounds/?utm_source=chatgpt.com (accessed 2025-09-28).

(12) Lu, S.; Yang, F.; Hu, H.; Li, D.; Hu, H.; Wang, J.; Duan, F.; Du, M. Isomeric Bipyridine-Based Covalent Organic Frameworks for Efficient Electrocatalytic Nitrate Reduction to Ammonia. *Journal of Materials Chemistry A* 2025, *13*, 9265–9273.

(13) Hu, H.; Miao, R.; Yang, F.; Duan, F.; Zhu, H.; Hu, Y.; Du, M.; Lu, S. Intrinsic Activity of Metalized Porphyrin-Based Covalent Organic Frameworks for Electrocatalytic Nitrate Reduction. *Advanced Energy Materials* 2024, *14* (6). https://doi.org/10.1002/AENM.202302608.

(14) Yang, J.; Qi, H.; Li, A.; Liu, X.; Yang, X.; Zhang, S.; Zhao, Q.; Jiang, Q.; Su, Y.; Zhang, L.; Li, J. F.; Tian, Z. Q.; Liu, W.; Wang, A.; Zhang, T. Potential-Driven Restructuring of Cu Single Atoms to Nanoparticles for Boosting the Electrochemical

Reduction of Nitrate to Ammonia. *Journal of the American Chemical Society* 2022, *144*, 12062–12071.

(15) Lv, F.; Sun, M.; Hu, Y.; Xu, J.; Huang, W.; Han, N.; Huang, B.; Li, Y. Near-Unity Electrochemical Conversion of Nitrate to Ammonia on Crystalline Nickel Porphyrin-Based Covalent Organic Frameworks. *Energy and Environmental Science* 2022, *16*, 201–209.

(16) Ouyang, L.; Yue, L.; Liu, Q.; Liu, Q.; Li, Z.; Sun, S.; Luo, Y.; Ali Alshehri, A.; Hamdy, M. S.; Kong, Q.; Sun, X. Cu Nanoparticles Decorated Juncus-Derived Carbon for Efficient Electrocatalytic Nitrite-to-Ammonia Conversion. *Journal of Colloid and Interface Science* 2022, *624*, 394–399.

(17) Murphy, E.; Liu, Y.; Matanovic, I.; Guo, S.; Tieu, P.; Huang, Y.; Ly, A.; Das, S.; Zenyuk, I.; Pan, X.; Spoerke, E.; Atanassov, P. Highly Durable and Selective Fe- and Mo-Based Atomically Dispersed Electrocatalysts for Nitrate Reduction to Ammonia via Distinct and Synergized NO2- Pathways. *ACS Catalysis* 2022, *12*, 6651–6662.

(18) Murphy, E.; Liu, Y.; Matanovic, I.; Guo, S.; Tieu, P.; Huang, Y.; Ly, A.; Das, S.; Zenyuk, I.; Pan, X.; Spoerke, E.; Atanassov, P. Highly Durable and Selective Fe- and Mo-Based Atomically Dispersed Electrocatalysts for Nitrate Reduction to Ammonia via Distinct and Synergized NO2- Pathways. *ACS Catalysis* 2022, *12*, 6651–6662.

(19) Wu, Z. Y.; Karamad, M.; Yong, X.; Huang, Q.; Cullen, D. A.; Zhu, P.; Xia, C.; Xiao, Q.; Shakouri, M.; Chen, F. Y.; Kim, J. Y. (Timothy); Xia, Y.; Heck, K.; Hu, Y.; Wong, M. S.; Li, Q.; Gates, I.; Siahrostami, S.; Wang, H. Electrochemical Ammonia Synthesis via Nitrate Reduction on Fe Single Atom Catalyst. *Nature Communications* 2021, *12*, 2870.

(20) Xu, T.; Ma, D.; Li, T.; Yue, L.; Luo, Y.; Lu, S.; Shi, X.; Asiri, A. M.; Yang, C.; Sun, X. Enhanced electrocatalytic N 2-to-NH 3 fixation by ZrS 2 nanofibers with a sulfur vacancy *Chemical Communications* 2020, *56*, 14031–14034.

(21) Parthasarathy, A.; Srinivasan, S.; Appleby, A. J.; Dhanda, A.; Pitsch, H. The Platinum Microelectrode/Nafion Interface: An Electrochemical Impedance Spectroscopic Analysis of Oxygen Reduction Kinetics and Nafion Characteristics Pressure Dependence of the Oxygen Reduction Reaction at the Platinum Microelectrode/Nafion Interface *Journal of the Electrochemical Society* 1992, *139*, 1634.
